# K16F/E22F Mutation Promotes Oligomerization and Alters β‑Sheet Topology of Aβ16–22 Peptides: Insights from Molecular Dynamics Simulations

**DOI:** 10.1021/acschemneuro.6c00003

**Published:** 2026-03-23

**Authors:** Viet Hoang Man, Xibing He, Taoyu Niu, Lianjin Cai, Fengyang Han, Phuong Nguyen, Junmei Wang

**Affiliations:** † Department of Pharmaceutical Sciences and Computational Chemical Genomics Screening Center, School of Pharmacy, 6614 University of Pittsburgh, Pittsburgh, Pennsylvania 15261, United States; ‡ Universite Paris Cite, CNRS, Laboratoire de Biochimie Theorique, 13 rue Pierre et Marie Curie, 75005 Paris, France

**Keywords:** amyloid-β peptides, K16F/E22F mutation, amyloid aggregation, oligomerization, β-sheet structure, molecular dynamics simulation

## Abstract

Amyloid-β (Aβ) aggregation into toxic oligomers and fibrils is a hallmark of Alzheimer’s disease. The Aβ_16–22_ fragment plays a critical role in the early stages of the aggregation of full-length Aβ peptides. Aggregation of Aβ_16–22_ is primarily driven by hydrophobic interactions within the LVFF core and electrostatic attraction between flanking residues K16 (+) and E22 (−). To dissect the relative contributions of these forces, we introduced a K16F/E22F double mutation, which eliminates charged residues while enhancing hydrophobicity and aromaticity. This substitution provides a controlled system to evaluate how specific interactions influence aggregation behavior. Using a novel computational protocol, featuring a strategically designed 4-mer system, multiple independent and long-time scale trajectories, and specialized analysis, we directly tracked and comprehensively characterized the oligomerization process. The mutation significantly enhanced both intra- and intermolecular interactions, promoting aggregation. It also altered the oligomerization pathways, as reflected in the distinct distribution across ten possible states formed by four Aβ_16–22_ peptides. Furthermore, while the wild-type peptide predominantly formed antiparallel β-sheets, the mutant favored parallel and mixed β-sheet arrangements. These results indicated that increased hydrophobicity and aromaticity facilitate more stable and polymorphic aggregation pathways. Our findings highlight the dominant role of hydrophobic interactions in early-stage Aβ aggregation and emphasize the therapeutic potential of targeting hydrophobic hotspots, such as the LVFF core, while accounting for structural polymorphism rather than focusing solely on disrupting electrostatic interactions.

## Graphical abstract

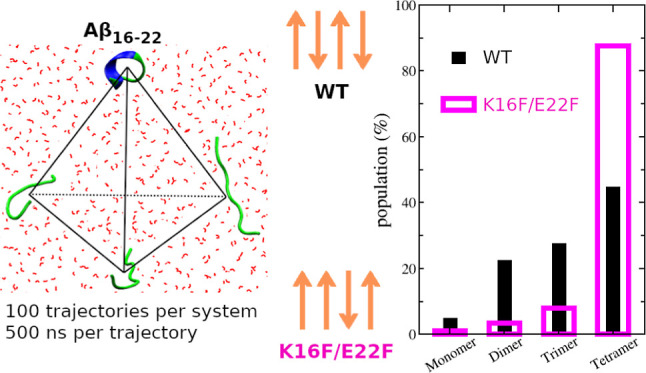

## Introduction

Amyloid aggregation is a biological process in which proteins misfold and aggregate into insoluble fibrils or plaques. The aggregation typically includes two phases, nucleation, followed by elongation. The nucleation phase, known as oligomerization, is characterized by the formation of soluble oligomers from monomers. The elongation phase is marked by the development of large prefibrillar and mature fibrillar structures. Amyloid aggregation plays a critical role in the pathology of several neurodegenerative disorders including Alzheimer’s disease (AD), Parkinson’s disease, and Huntington’s disease.^1–3^ The aggregation of amyloid-beta peptides and Tau proteins leads to the formation of insoluble fibrils, neurofibrillary tangles (NFT) of the Tau protein and Aβ senile plaques, which are two major hallmarks in the brain of Alzheimer’s Disease (AD) patients.^4^ Recent evidence show that both soluble oligomers and insoluble fibrils of Aβ peptides are neurotoxic, and the former is more harmful to the brain than the later.^5,6^ The soluble oligomers instigate multiple facets of AD neuropathology.^5,6^ Therefore, understanding the amyloid aggregation and its controlled factors is crucial to the therapeutic development for AD that is not curable yet.

Aβ peptides are proteolytic byproducts of the amyloid precursor protein. They occur in the brain with most common lengths of 40 (Aβ40) and 42 (Aβ42) amino acids. They have various physiological functions, such as acting as neuroprotectors, modulating synaptic activity, and potentially being essential for the survival of neuronal cells.^7–9^ The aggregation of Aβ peptides is strongly governed by many factors such as temperature, pH, monomeric concentration, and particularly intrinsic properties of peptides.^10–13^ Various mutations on Aβ peptides, which change the peptide intrinsic properties, have been found in familial AD. They include the English (H6R),^14^ Taiwanese (D7H),^15^ Tottori (D7N),^16^ E11K,^17^ K16N,^18^ K16Q,^19,20^ L17 V,^21^ Flemish (A21G),^22^ Dutch (E22Q),^23,24^ Italian (E22K),^25^ Arctic (E22G),^26^ Osaka (ΔE22, deletion),^27^ Iowa (D23N),^28^ L34 V,^21^ and A42T.^29^ Most of these mutations promote the aggregation of the peptides. Mireia Seuma, Ben Lehner, and Benedetta Bolognesi have performed a comprehensive and interesting experimental study that investigated the impact of substitutions, insertions, deletions, and truncations on amyloid beta fibril nucleation.^29^ They considered all possible single mutations on Aβ42 and measured the nucleation scores for the mutations. Their result showed that most mutations in the 30–41 residue region gave low nucleation scores, while the mutations on other residue regions had various nucleation scores depending on the mutated amino acid.

Atomistic simulations offer a detailed perspective on structures and dynamics of biomolecular system, complementing experimental work.^30–37^ With advances in algorithms and increased computational power, the scope of observable events has expanded significantly. The simulations provide critical insights that are often beyond the reach of traditional experiments, such as characterization of the unfolded state or the transiently populated intermediates that occur during complex binding and recognition events.^38–42^ Molecular dynamics (MD) simulations, in particular, have been extensively used to study amyloid aggregation. It has been used to uncover Aβ oligomerization pathways,^43^ capture the divergences of oligomeric structures,^44^ and investigate kinetics of the oligomerization at atomic resolution.^45–47^

Aβ_16–22_ (KLVFFAE) peptide, a fragment of Aβ40/Aβ42 and containing the central hydrophobic core, consists of 7 residues, two opposite charge residues at N-terminal (K16) and C-terminal (E22) and five hydrophobic residues at middle with two consecutive PHE residues (19 and 20). It aggregates and forms fibrils in vitro with the antiparallel ordering of the β-strands.^48^ It can help explore fundamental aspects of the thermodynamics and kinetics of amyloid aggregation.^49–54^ Thus, it is one of the most popular peptides used in amyloid aggregation studies. Nilsson et al. experimentally investigated the effects of aromatic, hydrophobic, and steric factors on the self-assembly of the peptides by performing different mutations at residues F19 and/or F20.^52,53^ Tycko et al. applied solid-state NMR method to reveal a pH-dependent antiparallel β-sheet registry in fibrils formed by Aβ_16–22_ peptides.^55^ Many computational studies used Aβ_16–22_ peptide and its variant mutations to capture atomistic mechanism of amyloid aggregation as well as the factors controlling the process.^46,50,54^

Aggregation of Aβ_16–22_ is primarily driven by hydrophobic interactions within the LVFF core and by electrostatic attraction between flanking residues K16 (+) and E22 (−). Several pathogenic mutations have been identified at these positions, including K16N,^18^ K16Q,^19,20^ E22Q,^23,24^ E22K,^25^ E22G,^26^ and the deletion mutation ΔE22.^27^ On the other hand, K16F and E22F mutations, although not found in natural Aβ sequences associated with Alzheimer’s disease, have been intentionally introduced in prior studies to enhance aggregation propensity and to investigate the role of hydrophobic and aromatic residues to amyloid aggregation.^56–58^ In this work, we aim to elucidate how specific physicochemical changes drive the Aβ_16–22_ self-assembly. To this end, we introduce the K16F/E22F double mutation, which replaces the charged Lys16 and Glu22 residues with phenylalanine, an aromatic amino acid, thereby increasing hydrophobicity and aromaticity while eliminating terminal charges. We hypothesize that these changes reduce peptide polarity and fundamentally alter early-stage intermolecular interactions. We employ a recently developed simulation protocol that combines innovative system design with extensive sampling across one hundred independent long-time scale trajectories. This approach enables direct tracking of early-stage oligomerization kinetics while ensuring satisfactory sampling convergence.^54,59^ Through computational investigation of the K16F/E22F mutant, we seek to determine how these physicochemical modifications govern the early-stage self-assembly of Aβ_16–22_ peptides. These insights improve our understanding of how sequence-dependent interactions influence early oligomerization behavior in amyloidogenic peptides and may provide useful context for future studies aimed at modulating aggregation in this region.

## Materials and Methods

### System Design

In this work, we examined the impact of double mutation K16F/E22F on the oligomerization of Aβ_16–22_ (sequence ACE-KLVFFAE-NME) peptides. We applied a 4mers system introduced in our previous studies to study the oligomerization of amyloid peptides.^54,59^ The system includes four monomers placed at the four vertices of a regular tetrahedron with a size of 3.5 nm, and the minimum distance between peptides is larger than 2 nm. We used four peptides in a simulation system to study the formation of oligomers up to the level of tetramer, which has been reported as one of the most toxic sizes of Aβ,^60,61^ while maintaining a computationally tractable system for large-scale simulations. In addition, arranging the four peptides in a regular tetrahedral geometry allows the construction of a homogeneous system in a truncated octahedral box under periodic boundary conditions. This approach minimizes initial structural bias and enables direct tracking of early-stage oligomerization kinetics. The four peptides then were put at the center of a truncated octahedral box, which has the volume of 409 nm^3^ and was solvated by 12255 water molecules (Figure 1a). It resulted in peptide concentrations of around 16.2 mM. Cl^–^ and Na^+^ ions were added into the system to have NaCl salt concentration of 0.15 M. For sampling, we constructed 100 different four-peptide systems by randomly selecting monomers from a monomeric database of 5000 monomers. To obtain the monomeric databank, we carried out a 100 ns NPT MD simulation of a monomer in explicit solvent and selected the 5000 monomeric structures from the last 50 ns. Then, the 100 4mers systems were used as the initial structures of the subsequent 100 independent MD simulations.

**Figure 1:**
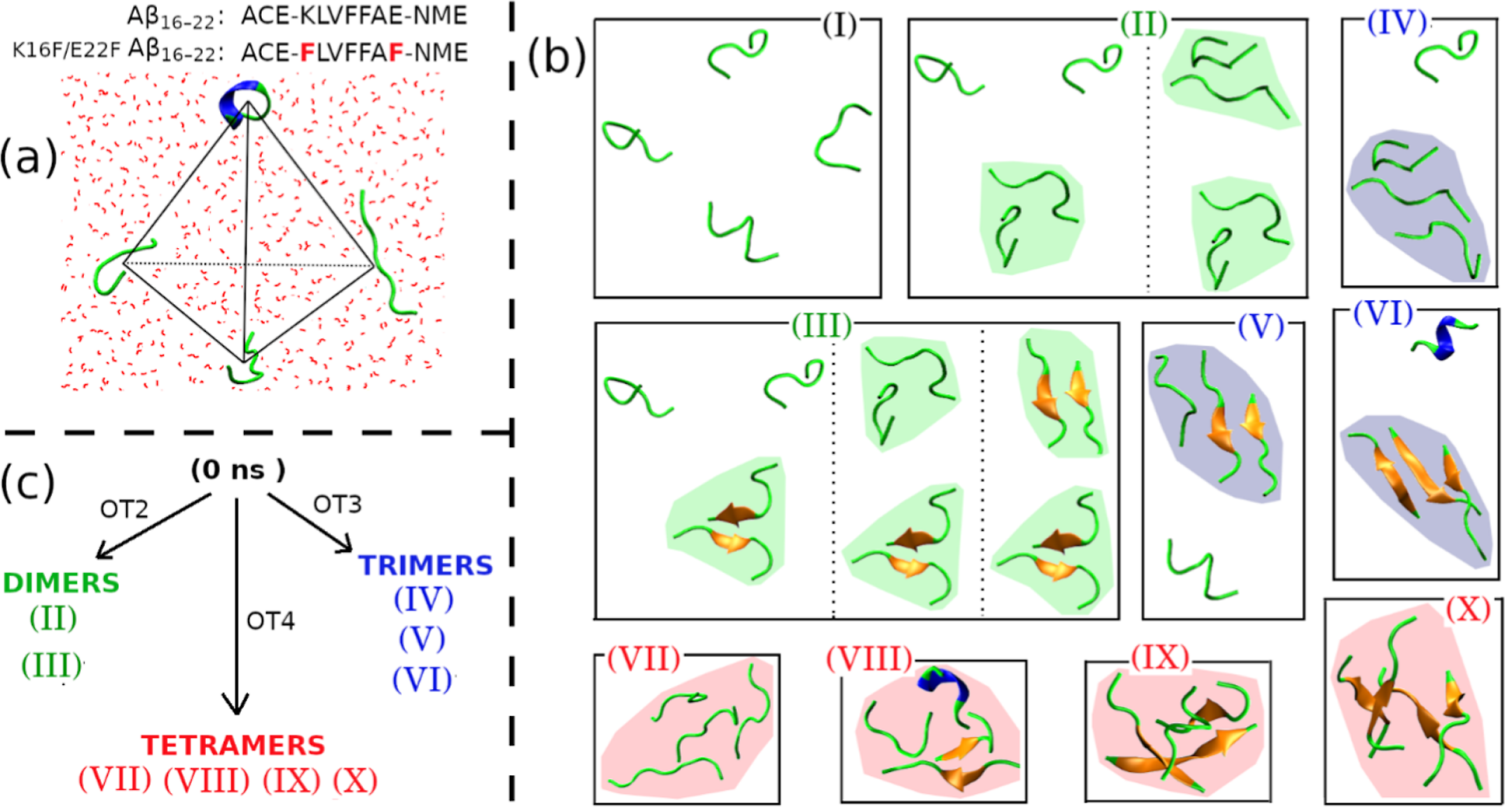
Definitions of the 4mers simulation systems (a) and the cartoon representation of 10 oligomeric states formed by four Aβ_16–22_ peptides (b). Schematic representation of oligomeric time is shown in (c), with OT2, OT3, and OT4 being dimeric time, trimeric time, and tetrameric time, respectively.

## Simulation Details

All the MD simulations were carried out using the pmemd.cuda module of the AMBER 18 software package.^62^ The ff14SB force field^63^ and TIP3P water model^64^ were used to model protein and the explicit solvent, respectively. Periodic boundary conditions were applied to minimize finite-size effects and approximate a bulk solution environment by effectively extending the simulation box in all directions. A long-range Coulombic interaction was evaluated by means of the Particle-Mesh Ewald method^65^ with a 1.0 nm cutoff. The van der Waals interactions were calculated by means of 1.0 nm atom-based nonbonded lists, with continuous corrections applied to the long-range parts. The constant pressure simulations were carried out at 1 atm via the Berendsen barostat^66^ with pressure relaxation time τ_p_ = 3.0 ps. Each system underwent the following steps. First, the steepest descent minimization followed by a conjugate gradient minimization with the peptide atoms fixed at their initial positions and then unrestrained steepest descent minimization and conjugate gradient minimization were carried out sequentially. After the system was minimized, a short MD simulation under a constant volume was first performed to heat up the system from 0 to 310 K with weak restraints on the protein atoms. Next, a Langevin dynamics at constant temperature (310 K) and constant pressure (1 atm) were applied for 100 ps (ps), after which the density of the system was found to be stable around 1.0 g/cm^3^. Finally, in the sampling phase, a 500 ns constant volume MD run at 310 K was performed using the leapfrog algorithm with a time step of 2 fs (fs). The temperature was regulated using Langevin dynamics with a collision frequency of 1 ps^–1^. The SHAKE algorithm^67^ was applied to all bonds involving hydrogen atoms. Conformations were saved every ten ps for postanalysis. In total, 50,000 snapshots were saved for postanalysis for each simulation system.

### Data Analysis

The secondary structure contents classified into β, helix, turn, and random coil were calculated by using the STRIDE algorithm.^68,69^ Here, the helix content includes 3–10 helix, Pi helix, and α-helix, the β content consists of extended residues, and the rest is random coil. CPPTRAJ tools^70^ was used to calculate the solvent-accessible surface area (SASA), gyrate (*R*_g_), and distances. The LCPO algorithm^71^ was applied for the SASA calculation. The intermolecular residue–residue interaction map was constructed using a 0.45 nm cut off for the distance between residues. The distance between the two residues is the smallest distance of any atom of the first residue to any atom of the second residue.

### Free-Energy Landscape (FEL)

The free-energy surface along the N-dimensional reaction coordinated V = (V_1_...,V_*N*_) is given by Δ*G*(V) = −*k*_B_T­[ln *P*(V)-ln *P*_max_], where *P*(V) is the probability distribution obtained from a histogram of MD data. *P*_max_ is the maximum of the distribution, which is subtracted to ensure that Δ*G* = 0 for the lowest-free-energy minimum. The *k*_B_ and T are the Boltzmann constant and simulation temperature, respectively. In this study, we used the sum of end-to-end distances (e2e) of the four peptides and the sum of distances between the center of mass of any peptide pair (cd) as reaction coordinates for the two-dimensional FEL.

The intermolecular interaction energy (IIE) of an 4-peptides complex is calculated by the following equation: $IIE=E_{complex}-\sum_{i=1}^{4}E_{i}$ . where *E*_complex_ and *E*_*i*_ denote the total energy of the peptide complex and the energy of peptide *i*, respectively. All energies were obtained from single-point energy calculations performed with the *pmemd* MD engine in AMBER, using the input parameters *imin* = 1, *maxcyc* = 0, and *ntf* = 1. Under this calculation protocol, bonded energy terms cancel out; consequently, the calculated IIE reflects only nonbonded contributions, namely, van der Waals and Coulombic interaction energies.

To investigate the oligomerization pathway of Aβ_16–22_ peptides, we classified the four Aβ_16–22_ peptides into ten states based on the oligomeric and β-sheet formation of the peptides (Figure 1b). The oligomers were defined by the distance between the peptides. Two peptides contact if their distance is smaller than or equal to 0.3 nm. The distance between two peptides is the smallest distance of any atom of the first peptide to any atom of the second peptide. A dimeric oligomer is formed when two monomers are in a contact state. Three monomers would become a trimeric oligomer when a dimer is formed and at least one peptide of the dimer is in a “contact state” with the third monomer. A tetrameric oligomer would be established when a trimeric oligomer is formed and at least one peptide of the trimer is in “contact state” with the fourth monomer. In state I, the four peptides do not contact each other, resulting in there not being any oligomer in this state. In state II, there are only dimeric oligomers and no β-sheet formation. In state III, the largest oligomeric size is dimer, and there is a dimer formed β-sheet. In state IV, a trimeric oligomer is formed by no β-sheet formation. In state V, there is a trimeric oligomer with two peptides in the β-strand structure. State VI contains a trimeric oligomer with all three peptides in the β-strand structure. In the states VII, VIII, IX, and X, the four peptides formed a tetrameric oligomer, but the number of β-strands is different between the states (Figure 1b). The transitions between the states were tracked and represented by lines between them. The oligomeric times, which are counted as the time when the first corresponding oligomer forms, were also measured (Figure 1c).

## Results and Discussion

In this work, we used conventional MD simulations to model the aggregation of Aβ_16–22_ peptides. Unlike some enhanced conformational sampling techniques such as replica exchange MD^72^ or simulated tempering,^73^ conventional MD simulation allows for tracking the evolution of the aggregation process directly. However, with conventional MD simulations, the system is often trapped in one of many local minima, resulting in only a single pathway and its associated kinetics being observed. Consequently, the full picture of aggregation may not be accurately represented. To overcome the limitation of conventional MD simulations and achieve convergence of conformational sampling, we carried out multiple long MD simulations with different initial structures. For each system, we sampled one hundred 500 ns MD simulation trajectories. Our simulations reached equilibrium states after 200 ns (Figures S1 and S2 in Supporting Information). Thus, we used the data collected from the last 300 ns of the MD trajectories for most statistical analyses. To assess the convergence of sampling, we compared the distributions of three reaction coordinates: the IIE, the radius of gyration (*R*_g_), and the solvent-accessible surface area (SASA). The distributions of these reaction coordinates are presented in Figure S3 (in Supporting Information) for three ensemble statistics: the last 300 ns (from 200 to 500 ns) of all 100 trajectories, 220 ns spanning from 200 to 420 ns of all 100 trajectories, and the last 300 ns of 75 randomly selected trajectories. Notably, the three ensemble statistics yielded very similar distributions for all three of the reaction coordinates. These findings provide strong assurance that our sampling strategy, 100 independent 500 ns MD runs, achieves sufficient and converged sampling, enabling us to discuss the impact of the mutation on the oligomerization of Aβ_16–22_ peptides.

### K16F/E22F Mutation Promotes the Oligomerization of Aβ16–22 Peptides

To assess the impact of the double mutations on the oligomerization of Aβ_16–22_ peptides, we first considered general structural parameters including *R*_g_, SASA, intermolecular residue–residue interaction (*N*_inter_), and intramolecular residue–residue interaction of the peptides (*N*_intra_). Figure S1 shows the time course of *R*_g_, SASA, *N*_inter_, and *N*_intra_ along the MD simulation time. As seen, those parameters dramatically changed in the first 50 ns and fluctuated around the equilibrium values in the last 300 ns. The result demonstrated that the mutation leads to decreasing *R*_g_ and SASA and increasing *N*_intra_ and *N*_inter_. The average of the structural parameters in the last 300 ns is listed in Table 1. The values of *R*_g_ and SASA of mutated peptides were 1.0 ± 0.1 and 30 ± 1 nm^2^, respectively, much smaller than the corresponding values of 1.5 ± 0.2 and 33 ± 2 nm^2^ of the wild-type Aβ_16–22_ peptides. The values of *N*_intra_ and *N*_inter_ were 29 ± 1 and 34 ± 5 for the wild-type peptides and 32 ± 1 and 45 ± 2 for the mutated peptides. We also constructed free-energy landscapes using two reaction coordinates: total end-to-end distances (e2e) and total center-to-center distances (cd) of all peptide pairs (Figure 2a,b) and intermolecular residue–residue interaction maps (Figure 2c,d). The FELs of the wild-type system had three minima, while the mutated system had two. To obtain the centers and populations of the minima, we performed k-mean clustering analysis of the FELs data. The three minima of the wild-type system were located at (5.6,7.0), (5.3,14.1), and (51.,19.1), with corresponding population sizes of 55%, 27%, and 18%. The two minima of the mutated system had centers at (5.3,6.6) and (4.8,15.0), and their population sizes were 94.5% and 5.5%. The FELs clustering analysis implied that the wild-type peptides were more extended and more distant from each other than the mutated ones. The residue–residue contact maps show that intermolecular interaction frequencies are generally higher in the mutated system than in the wild-type system. In the contact map of the wild-type peptides, interaction frequencies for residue pairs along the main diagonal are consistently higher than those along the antidiagonal, indicating a preference for antiparallel peptide arrangements. In contrast, the contact map of the mutant peptides exhibits a more balanced distribution of interaction frequencies between the two diagonals, implying a greater diversity of relative peptide orientations and a reduced preference for a specific registry.

**Table 1:** Values of the Overall Structural Parameters Averaged from the Last 300 ns of MD Simulations^a^

system	β (%)	helix (%)	turn (%)	coil (%)	SASA (nm^2^)	*R*_g_ (nm)	*N*_inter_	*N*_intra_	IIEvdw (kcal/mol)	IIEele (kcal/mol)
WT	10 ± 3	9 ± 2	35 ± 3	46 ± 3	33 ± 2	1.5 ± 0.2	34 ± 5	29 ± 1	–52 ± 7	–110 ± 19
K16F/E22F	9 ± 3	15 ± 3	37 ± 4	39 ± 4	30 ± 1	1.0 ± 0.1	45 ± 2	32 ± 1	–76 ± 5	–40 ± 6

a The averaged values of secondary structures (β, helix, turn, and coil contents), SASA, radius of gyration (*R*_g_), number of intermolecular residue–residue interaction (*N*_inter_), number of intramolecular residue–residue interaction (*N*_intra_), intermolecular van der Waals interaction energy (IIEvdw) in kcal/mol, and intermolecular Coulombic interaction energy (IIEele) in kcal/mol.

**Figure 2:**
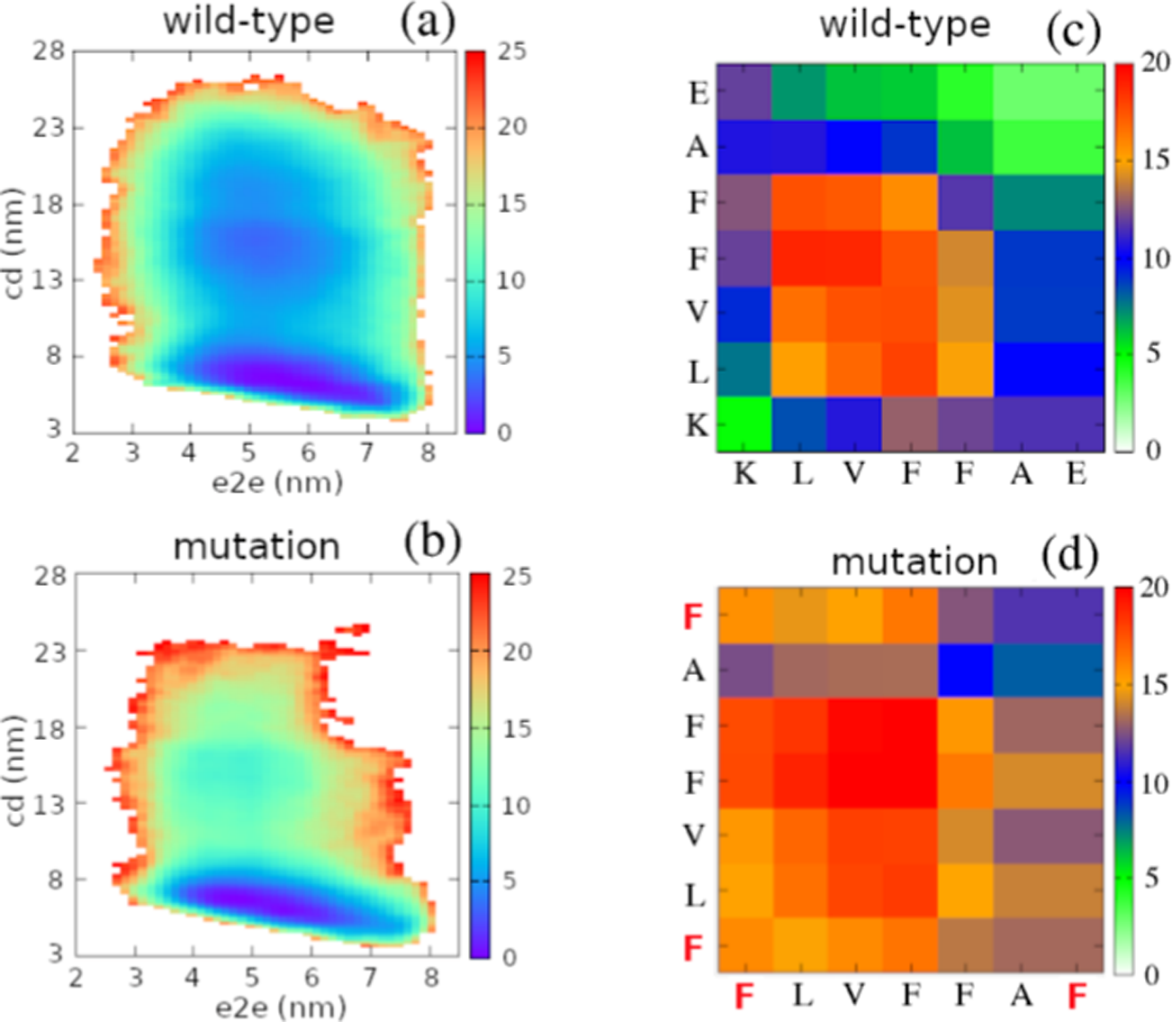
Free-energy landscapes (a,b) and intermolecular residue–residue interaction maps (c,d) for the wild-type and K16F/E22F mutant systems. The free-energy landscapes are projected onto two reaction coordinates: the end-to-end distances (e2e) of the four peptides and the sum of center-of-mass distances between all peptide pairs (cd). In the interaction maps, residues are labeled using the one-letter codes of the corresponding amino acids.

Next, we tracked oligomerization time and the population of monomer and oligomers along the simulation time (Figure 3). Here, the term of oligomerization time means the first time an oligomer is formed from the starting time of the simulation. The dimeric, trimeric, and tetrameric oligomeric time were 2 ± 1 ns, 10 ± 3 ns, and 32 ± 7 ns for the wild-type system, while they were 2 ± 1 ns, 7 ± 2 ns, and 18 ± 4 ns for the mutated system, respectively. The monomeric concentration in both systems rapidly decreased in the first 20 ns of the simulation time (Figure 3a). It reached zero after 50 ns in the mutated system, while it fluctuated around 5% after 50 ns in the wild-type system. This result indicated that the aggregation was very fast for both systems. This was expected because the amyloid peptide concentration of the simulation systems was in the mM range.^46,59^ The fast oligomerization time at the nanosecond scale imposes a grand challenge for current experimental techniques to observe the event. The concentration of dimeric species in both systems demonstrated a common pattern that the concentration was rapidly increased, reaching the maximum around 5–10 ns and equilibrium after 100 ns. After reaching equilibrium, the dimeric concentration was almost reduced to zero in the mutated system, while it was around 10–20% in the wild-type system. For the trimeric species, the time course of the concentration is similar to that of the dimeric species in the mutated system. In contrast, in the wild-type system, the concentration of trimetric species was first increased in 0–50 ns, fluctuated in 50–150 ns, and reduced equilibrium thereafter with the slightly decreased concentration. The evolution of the tetrameric concentration in both systems was similar, with the concentration being steadily increased and reaching equilibrium after 100 ns for the mutated system and 200 ns for the wild-type system. Another striking difference lies that the tetrameric concentration after equilibrium was increased to 100% in the mutated system, while it was only 60% in the wild-type system. The results clearly indicated that the mutation can promote oligomerization as the oligomerization time and the growth of oligomers in the mutated system are accelerated.

**Figure 3:**
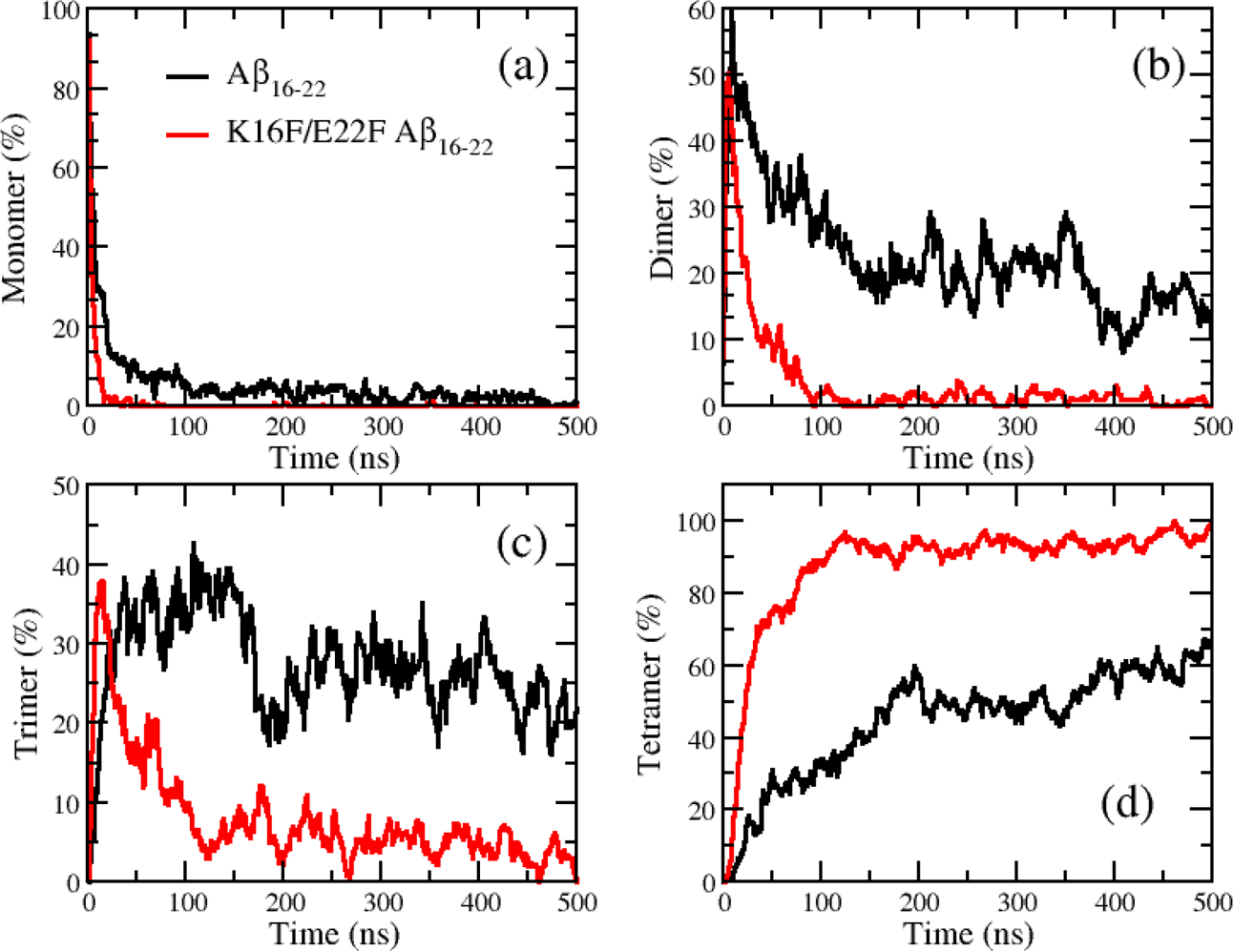
Time evolution of the populations of monomers (a), dimers (b), trimers (c), and tetramers (d) for the wild-type (black lines) and K16F/E22F mutant (red lines) Aβ_16–22_ peptides.

### K16F/E22F Mutation Induces π–π Interactions of Aβ_16–22_ Peptides

In the early-stages of Aβ oligomerization, π–π interactions play a critical role as primary driving forces for the initial self-assembly of Aβ monomers, particularly within hydrophobic regions involving phenylalanine residues.^74–76^ These interactions stabilize early oligomeric species and provide structural order and directionality during the Aβ assembly, ultimately leading to the formation of mature fibrils. Given their essential role in early oligomerization, π–π interactions represent attractive targets for potential therapeutic intervention.^77–79^ The K16F/E22F mutation introduces additional phenylalanine residues into the Aβ peptide. Therefore, it is of great interest to examine mutation-induced π–π interactions. Owing to the diversity of possible π-stacking geometries, π–π interactions between pairs of phenylalanine residues were quantified by calculating the binding energies between their aromatic rings using the MMGBSA method.^80^ Figure 4 presents the average energies and populations of both intramolecular and intermolecular π–π interactions. The population of a π–π interaction is defined as the percentage of simulation frames in which the interaction energy between the aromatic rings is lower than −1 kcal/mol. In the wild-type peptide, two phenylalanine residues are present: PHE19 (F4) and PHE20 (F5). In contrast, the mutant peptide contains two additional phenylalanine residues: PHE16 (F1) and PHE22 (F7). For the wild-type system, one type of intramolecular π–π interaction (F4–F5) and three types of intermolecular π–π interactions (F4–F4, F4–F5, and F5–F5) are observed. By comparison, the mutant system exhibits six types of intramolecular and ten types of intermolecular π–π interactions (Figure 4). Notably, both intramolecular and intermolecular π–π interactions in the mutant system are significantly stronger than those in the wild-type system, as indicated by lower interaction energies. Consistent with this observation, the populations of these interactions, particularly intermolecular π–π contacts, are markedly higher in the mutant system. Collectively, these results indicate that the K16F/E22F mutation not only increases the number of π–π interaction pairs but also strengthens and promotes pre-existing π–π interactions, thereby enhancing intra- and intermolecular interactions.

**Figure 4:**
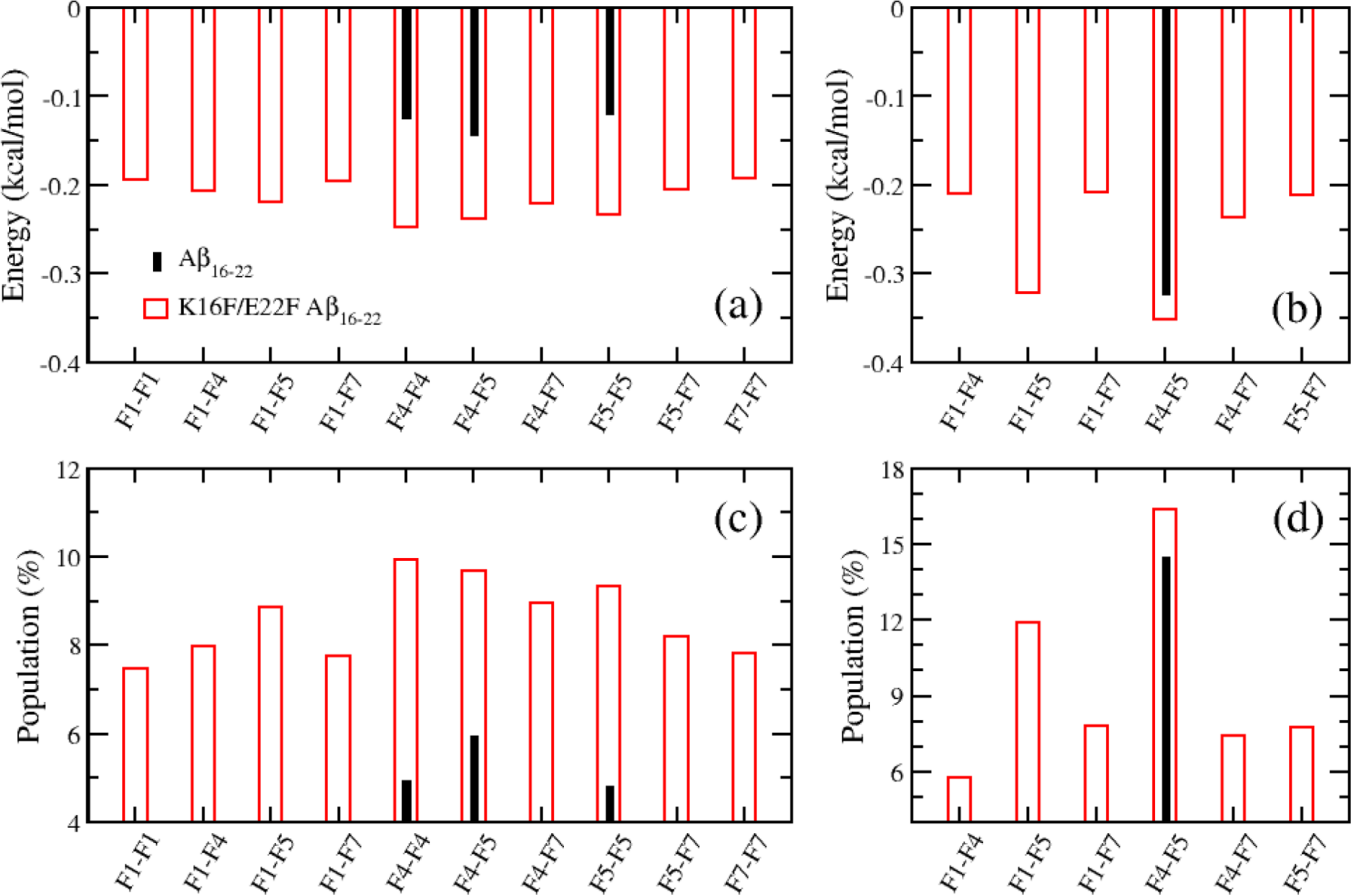
π–π interactions in Aβ_16–22_ peptides. Shown are the time-averaged interaction energies between pairs of phenylalanine residues (a,b) and the corresponding populations of π–π interactions (c,d). Panels (a) and (c) correspond to intermolecular phenylalanine interactions, whereas panels (b,d) correspond to intramolecular phenylalanine interactions. The interaction population is defined as the percentage of simulation frames in which the instantaneous π–π interaction energy between a given phenylalanine pair is lower than −1.0 kcal/mol, whereas the energies shown in panels (a,b) represent averages over the full trajectories and therefore span a narrower energy range. Black and red bars denote the wild-type and K16F/E22F mutant Aβ_16–22_ peptides, respectively.

### Impact of K16F/E22F Mutation on the Secondary Structures and β-sheet Formation of Aβ_16–22_ Peptides

Secondary structural contents and β-sheet formation are important characteristics of amyloid aggregation. In this section, we study the impact of the double mutation on those factors. The secondary structural profile along residue sequence is shown in Figure 5, and the time dependence of the structural contents is present in Figure S2. The averages of the secondary structural contents from the last 300 ns of wild-type and mutated peptides are summarized in Table 1. The secondary structure distribution of the wild-type Aβ_16–22_ peptide is 10% β-sheet, 9% helix, 35% turn, and 46% coil. This distribution indicates a dominance of turn and coil structures in the early oligomerization states of small Aβ_16–22_ oligomers, in good agreement with prior MD studies.^81,82^ Early all-atom simulations by Favrin et al. demonstrated that monomers and small oligomers of Aβ_16–22_ predominantly adopt disordered conformations, with random coil and turn structures dominating the ensemble and only minor populations of β-sheet and helical contents.^81^ Similarly, Klimov and Thirumalai reported that early Aβ_16–22_ assemblies populate heterogeneous conformational ensembles, including transient helical states, prior to β-sheet stabilization during aggregation.^82^ For the mutant peptide, the secondary structure distribution is 9% β-sheet, 15% helix, 37% turn, and 39% coil. Compared to the wild type, the mutations have little effect on the β-sheet and turn contents but significantly alter the balance between helix and coil structures, resulting in an increased helical population accompanied by a corresponding decrease in coil content. This pronounced shift in helical and coil populations in the mutant peptide may be attributed to enhanced π–π interactions, which are known to stabilize transient ordered conformations and reduce conformational disorder in amyloidogenic peptides.^83–85^ In contrast, charged residues K16 and E22 in the wild-type peptide interact strongly with the solvent, favoring more flexible and disordered coil conformations.

**Figure 5:**
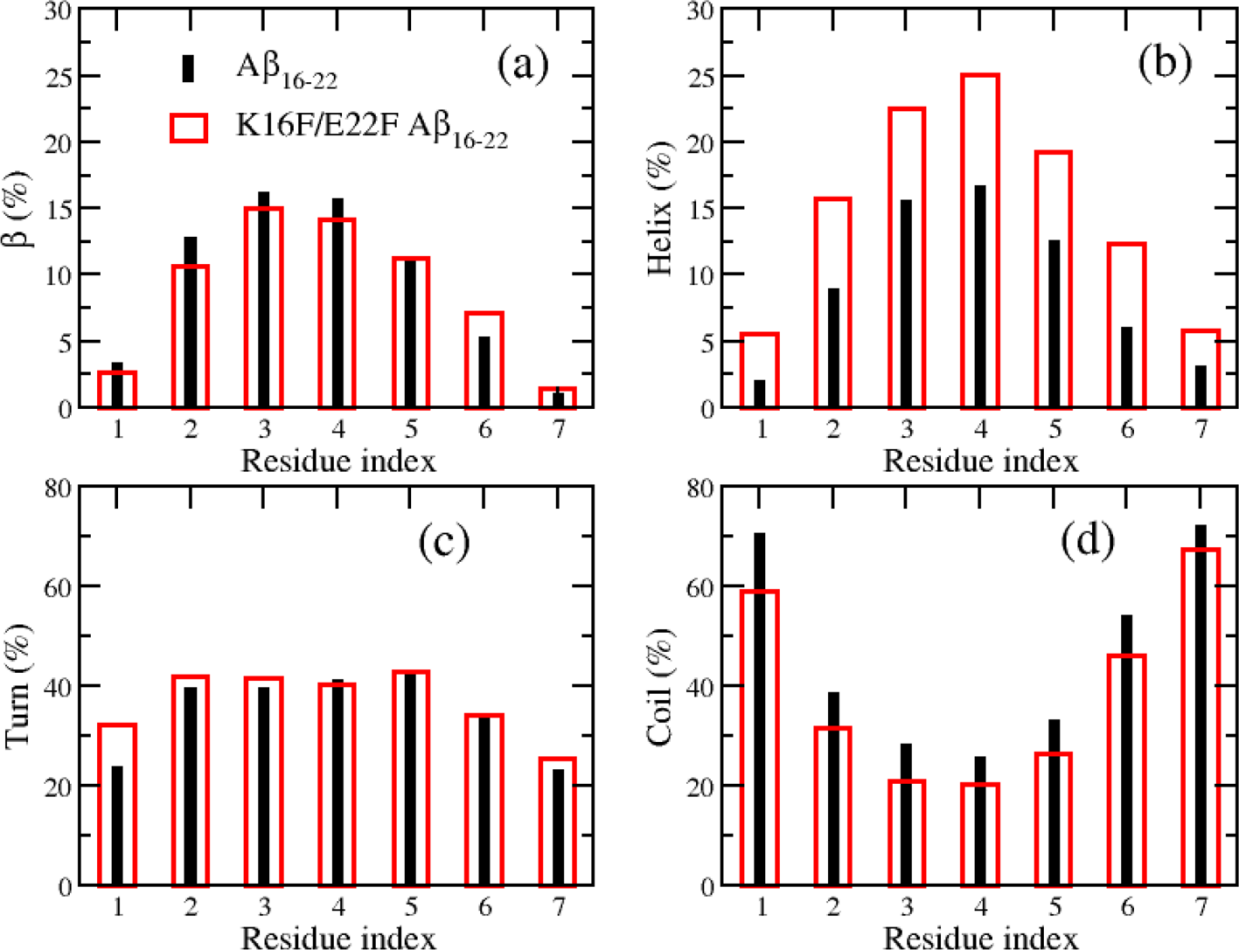
Secondary structural populations along the amino acid sequence of Aβ_16–22_ peptides, including β (a), helix (b), turn (c), and coil (d). The data were calculated using snapshots sampled in the last 300 ns of all 100 MD trajectories for each system.

Secondary structural transitions are a hallmark of amyloid aggregation and reflect the dynamic reorganization of peptide conformational ensembles during oligomerization. As shown in Figure 6 and Tables S1 and S2 (in Supporting Information), our analysis of secondary structural transitions along the Aβ_16–22_ sequence reveals frequent coil↔turn, coil↔helix, coil↔β, turn↔helix, and turn↔β transitions, whereas direct helix↔β transitions are not observed. The dominance of coil↔turn interconversions across most residues is consistent with the highly disordered nature of early Aβ assemblies reported in both experimental and simulation studies.^81,82,86,87^ Notably, strong turn↔helix transitions are observed for most residues, especially the central residues, of the peptide (Figure 6), suggesting that turn conformations can serve as intermediates that facilitate local helix formation. Such transient helical states have been reported previously in early Aβ oligomers and are thought to represent metastable intermediates that precede β-sheet stabilization during aggregation.^84,88,89^ The absence of direct helix↔β transitions in our simulations instead supports a multistep pathway in which helices first relax into coil or turn states before reorganizing into β-sheet structures, consistent with the commonly proposed helix→coil→β mechanism.^84,88^ Overall, these results indicate that Aβ_16–22_ aggregation proceeds through a network of interconnected secondary structure transitions dominated by disordered intermediates and transient helical states, rather than through a single direct conformational conversion pathway.^83,85,90^

**Figure 6:**
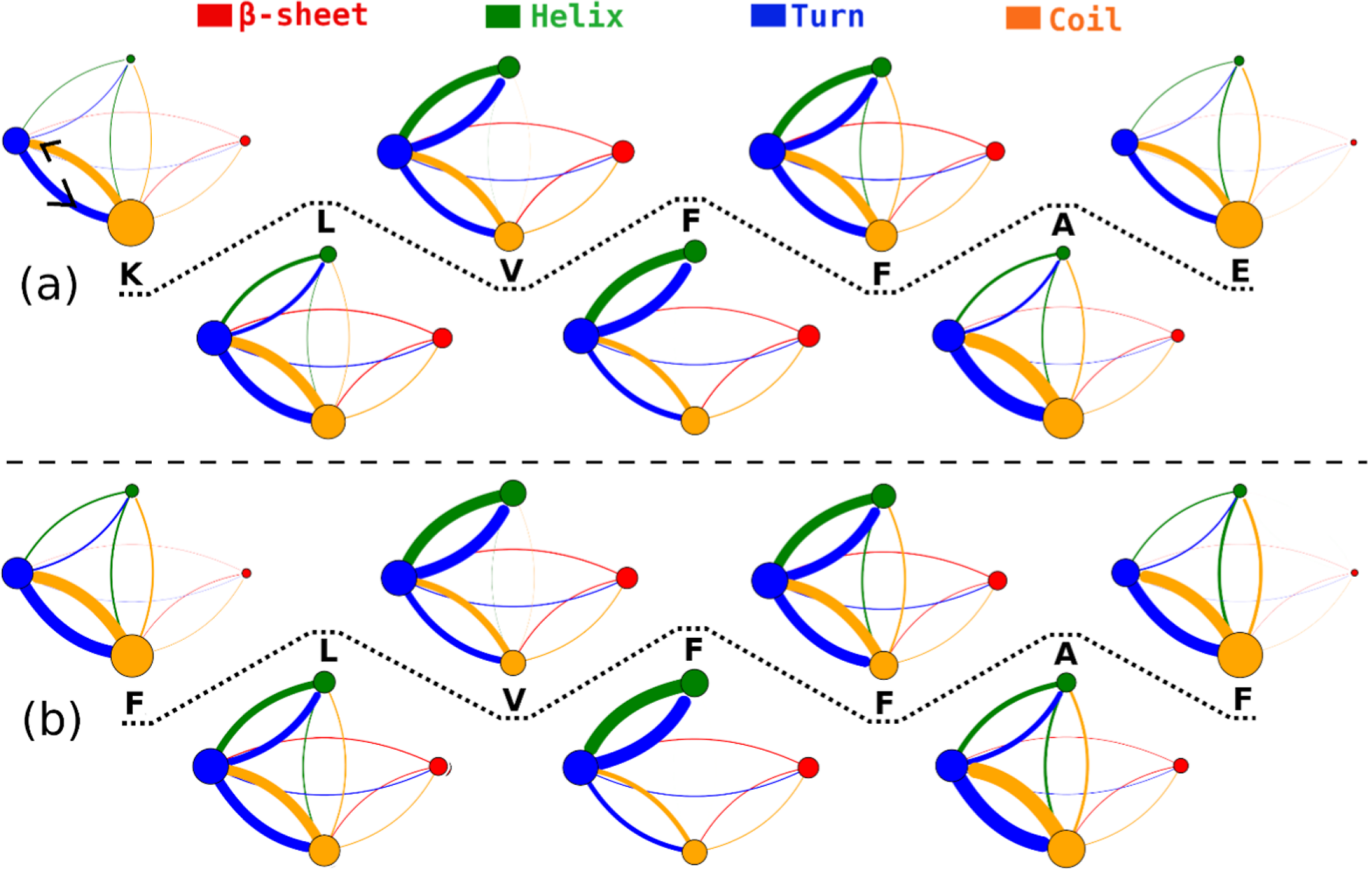
Secondary structural transitions along the amino acid sequence of wild-type (a) and K16F/E22F (b) Aβ16–22 peptides. Structural populations are represented by colored circles: red for β-sheet, green for helix, blue for turn, and orange for coil. Transitions between secondary structure states are depicted by curved lines, with colors matching those of the source structural populations. The population of each secondary structure and the corresponding transition frequency are proportional to the area of the circles and the thickness of the connecting curves, respectively. The data were calculated using snapshots sampled in the last 300 ns of all 100 MD trajectories for each system.

The detailed residue-specific secondary-structure transition frequencies for the wild-type and mutated Aβ_16–22_ peptides are summarized in Tables S1 and S2 (in Supporting Information). These data indicate that the K16F/E22F mutation alters the transition patterns across several residues. For the turn↔β transitions, the mutation decreases the transition frequencies at residues 16, 17, and 20, while slightly increasing those at residues 18, 21, and 22. For the coil↔β transitions, the changes follow a trend similar to those observed for the turn↔β transitions, except for residue 18. Notably, the mutation leads to higher frequencies of coil↔helix and turn↔helix transitions for most residues. These changes are consistent with the increased helical propensity observed in the mutant peptides relative to the wild-type peptides.

To examine the β-sheet formation of Aβ_16–22_ peptides, we classified β-sheets by oligomer sizes and β-strand types. The oligomer size includes dimer, trimer, and tetramer and the β-strand type include parallel, antiparallel, and mixed (containing both parallel and antiparallel). In total, we have defined eight different β-sheets, which are parallel dimer β-sheet (pBS2), antiparallel dimer β-sheet (apBS2), parallel trimer β-sheet (pBS3), antiparallel trimer β-sheet (apBS3), mix-trimer β-sheet (mBS3), parallel tetramer β-sheet (pBS4), antiparallel tetramer βsheet (apBS4), and mix-tetramer β-sheet (mBS4). All possible β-sheet formations of four Aβ_16_–_22_ peptides are demonstrated in Figure 7. The populations of dimer-β-sheet (pBS2, apBS2), trimer-β-sheet (pBS3, apBS3, mBS3), and tetramer-β-sheet (pBS4, apBS4, mBS4) were 24.4, 5.3, and 0.8% for the wild-type system and 27.4, 5.3, and 1.5% for the mutated system. It is interesting that the total β-sheet populations of the mutated system were slightly higher than that of the wild-type system, while the β-content of the mutated system was slightly smaller than that of the wild-type system. This inconsistency can be explained by the different ways to measure the β content and β-sheet. The β-content was calculated using the number of residues in the β state, while the β-sheet was recognized considering multiple ψ and ϕ torsional angles of amino acid residues in the sequence. Therefore, this result indicated that the extended strands in β-sheets were longer in wild-type peptides than those in the mutated peptides. We also calculated the relative population weights of the different β-sheet types with the same oligomer size. For the dimer, the population ratio of pBS2 and apBS2 was 3/7 for both the wild-type and mutated systems. For trimer, the population ratio of pBS3, apBS3, and mBS3 was 3/63/34 for wild-type peptides and 20/42/38 for the mutated species. For tetramer, the population ratio of pBS4, apBS4, and mBS4 was 1/72/27 for wild-type peptides and 4/21/27 for mutated peptides. Those statistical results indicate that wild-type Aβ_16–22_ peptides preferred antiparallel β-sheet, while the mutated Aβ_16_–_22_ peptides favored mixed β-sheets.

**Figure 7:**
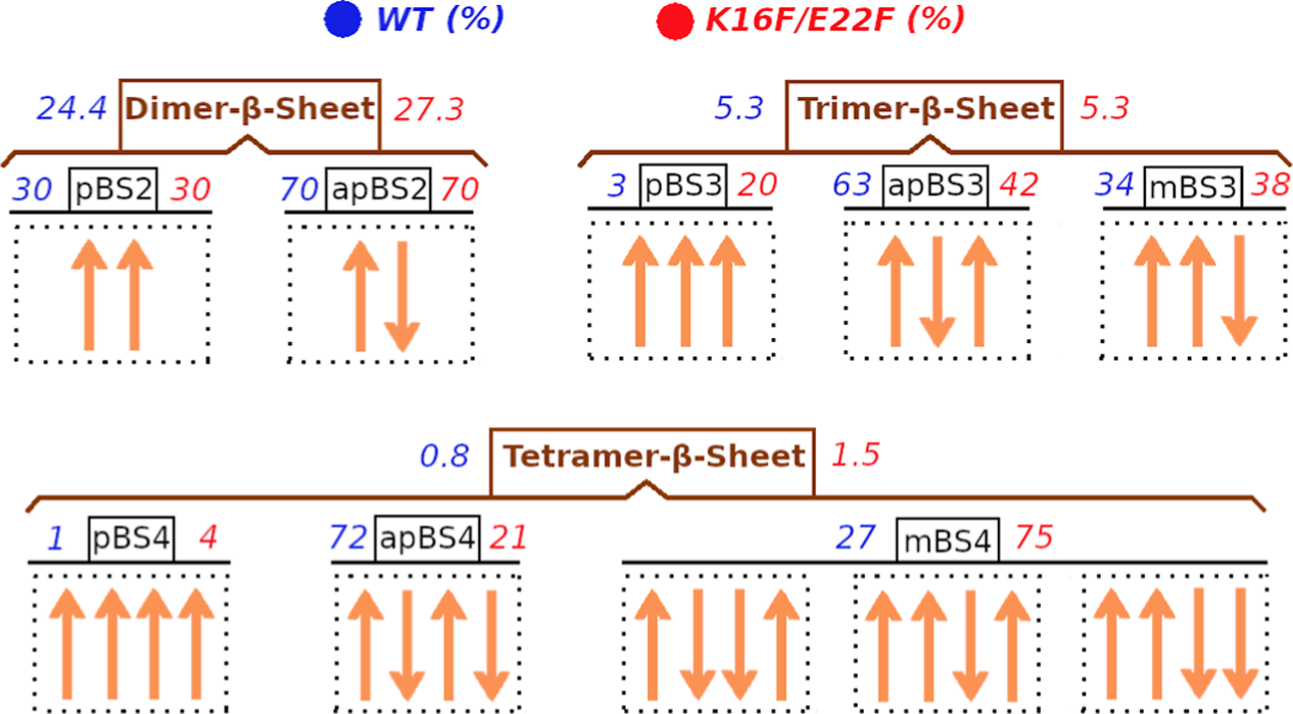
Populations of different β-sheet types, which include parallel dimer β-sheet (pBS2), antiparallel dimer β-sheet (apBS2), parallel trimer β-sheet (pBS3), antiparallel trimer β-sheet (apBS3), mix-trimer β-sheet (mBS3), parallel tetramer β-sheet (pBS4), antiparallel tetramer β-sheet (apBS4), mix-tetramer β-sheet (mBS4). The data were generated using snapshots collected from the 500 ns of all 100 MD trajectories for each system.

### Impact of K16F/E22F Mutations on the Aggregation Pathway of Aβ_16–22_ Peptides

As mentioned above, conventional simulation allows us to track state transitions directly. Therefore, this approach has advantages in investigating aggregation pathways. To reveal the oligomerization pathways, we defined 10 aggregation states formed by the four Aβ_16–22_ peptides, and we tracked the transition frequency between those states (Figure 1). The population sizes of the 10 states and the transition frequency between any two states are depicted in Figure 8 for both two systems. Although the population sizes of the 10 states were different between the two systems, there were some common features: first, state VII had the highest population among the ten states; second the population of the none-β state (II of dimer, IV of trimer, and VII of tetramer) is the largest among the states with the same oligomer size. This implies that the rearranging process of aggregation, in which peptides rearrange to form β-sheet, is much longer than the oligomerization process, where separated monomers approach each other to form oligomers. On the other hand, the populations of states I to VI in the wild-type system were higher than the corresponding ones in the mutated system, while the populations of states VII to X in the wild-type system were smaller than the related ones in the mutated system. This result again demonstrates the significant impact of the mutations on the oligomeric structures of the Aβ peptides. The transition frequency between two states varied, depending on the types of two states, as well as the wide-type or mutated system. The transitions can be classified into two types: oligomeric growth transition and oligomeric rearranging transition. An oligomeric growth transition occurs between two states with different oligomeric sizes, and an oligomeric rearranging transition occurs when the two states have the same oligomeric size. In the wild-type system, the oligomeric rearranging transitions including IV–V, VII–VIII, and VIII-IX had higher frequencies than other ones. However, oligomeric growth transitions (I–II, II–VI, III–V, and IV–VIII) also frequently occurred. In the mutated systems, tetrameric rearranging transitions, VII–VIII and VIII-IX, significantly outnumbered other types of rearranging transitions. The frequencies of oligomeric growth transitions in the mutated system were substantially lower than those in the wild-type system, indicating that oligomer growth proceeds more rapidly in the mutant. This behavior implies stronger intermolecular interactions among the mutated peptides, leading to more stable oligomeric assemblies. In contrast, the higher transition frequencies observed for the wild-type system suggest that its oligomers are more transient and undergo frequent association–dissociation events. This is expected since the mutation increases the hydrophobicity and reduces the polarity of Aβ_16–22_ peptide. Note that a polar solvent such as water can interfere with the interaction between polar molecules and promote the interaction between hydrophobic molecules. In conclusion, the double mutation dramatically changed the oligomerization pathways of Aβ_16–22_ peptides.

**Figure 8:**
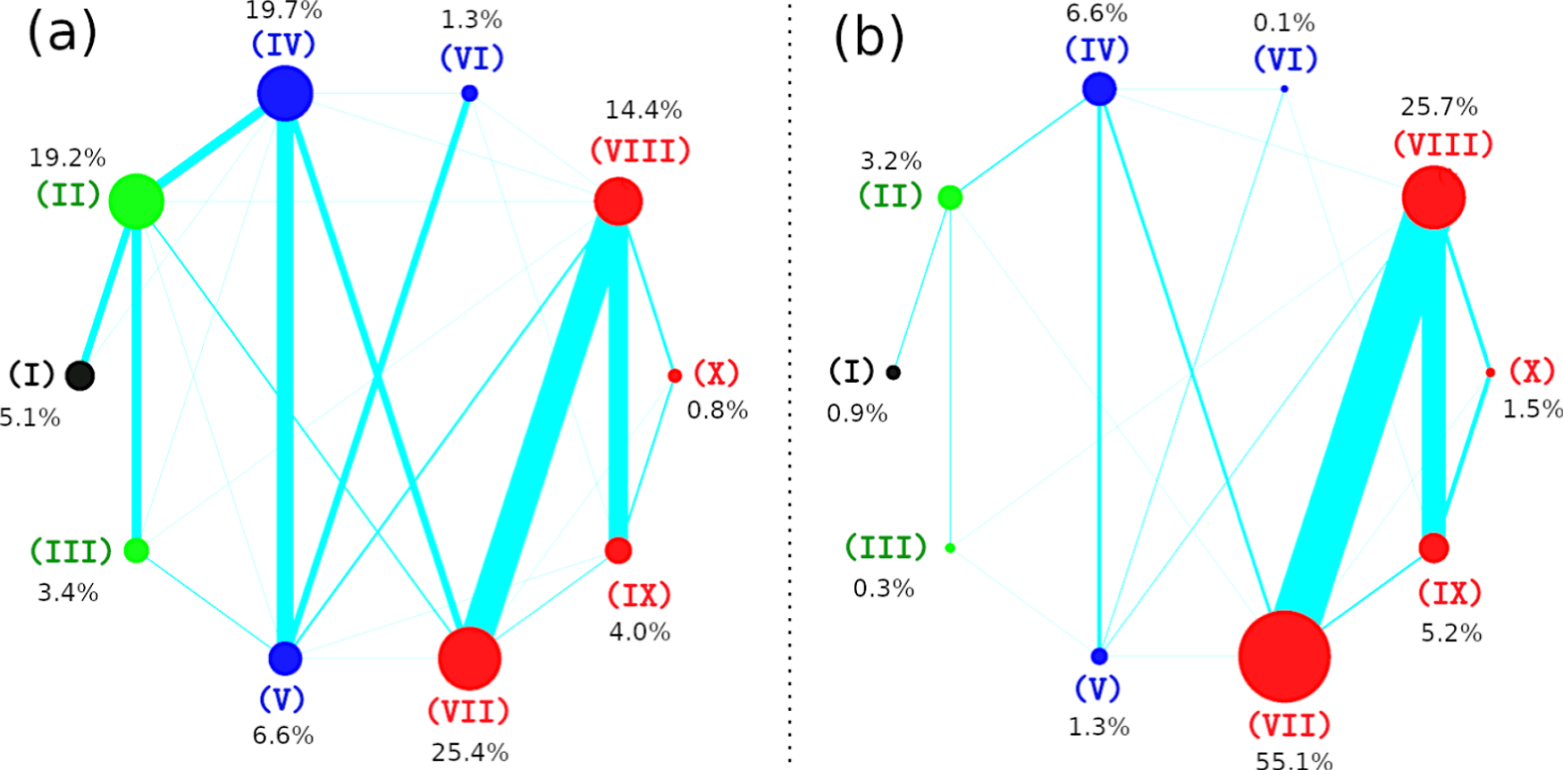
Population sizes of the 10 states and the transitions between the states in the wild-type system (a) and K16F/E22F mutation system (b). Each state’s population is represented by a circle, with the area of the circle proportional to the population size. The exact population values are indicated by black numbers. The frequency of transitions between two states is depicted by the thickness of the line connecting them. The data were calculated using snapshots collected from 500 ns of all 100 MD trajectories for each system.

The results presented above indicate that the K16F/E22F double mutation is associated with an overall increase in the helical content of Aβ_16–22_ peptides. To further examine how this increase in helicity relates to oligomerization, we analyzed the helical content as a function of oligomer size. For the wild-type system, the helical content remains nearly constant across oligomer sizes, with values of 9.38% for monomers, 9.52% for dimers, 9.31% for trimers, and 10.07% for tetramers. In contrast, the K16F/E22F mutant exhibits a noticeable dependence on the oligomer size, with helical contents of 10.27% (monomer), 9.70% (dimer), 13.28% (trimer), and 15.17% (tetramer). These data indicate that the increase in the helical content observed in the mutant is more pronounced in larger oligomeric species than in monomers or dimers. Notably, tetramers display higher helical content than monomers in both systems, with a stronger increase in the mutant. This trend is consistent with the oligomer-size distributions observed in the simulations, where tetramers form more rapidly and reach a higher population in the mutant system. During the last 300 ns of simulation, the populations in the wild-type system were 2.4% monomers, 18.6% dimers, 25.5% trimers, and 53.5% tetramers, whereas in the mutant system, they were 0% monomers, 1.2% dimers, 4.8% trimers, and 94.0% tetramers. These observations suggest that larger oligomers in the mutant system tend to sample conformations with higher helical content. Although the present simulations cannot directly establish a specific aggregation mechanism, the presence of transient helical segments within early oligomeric assemblies is consistent with previous studies suggesting that such intermediates may precede the formation of β-sheet–rich structures. For example, similar structural intermediates have been proposed for full-length Aβ42 peptides by Misra et al.^91^

### Robustness of Mutation-Induced Aggregation Trends across Force Fields

Force fields play a critical role in MD simulations, particularly in studies of amyloid.^36,38,44,46,47,92,93^ Our recent force-field benchmarking studies on amyloid aggregation, including tau fragment and Aβ_16–22_ peptides, demonstrated that aggregation behavior is highly sensitive to the choice of force field.^47,92^ These benchmarks identified AMBER ff14SB and CHARMM36m,^94^ two widely used force fields, as among the most suitable force fields for studying amyloid peptide assembly, as they provide a balanced description of both structural ensembles and aggregation kinetics.^47,92^ In contrast, recent force-field benchmarking studies of peptide folding using accelerated MD have reported that ff14SB exhibits a tendency to overstabilize helical.^95^ Consequently, it is important to assess whether the observed effects of the K16F/E22F mutation on Aβ_16–22_ peptides, particularly the increased helical content, persist when simulations are performed using the CHARMM36m force field and the modified TIP3 water model. To this end, we carried out additional simulations using CHARMM36m while keeping all other simulation parameters identical to those used in the ff14SB simulations. Although the detailed values obtained from the CHARMM36m simulations differ from those obtained with ff14SB, the mutation-induced trends remain consistent across both force fields. First, the distributions of the radius of gyration (*R*_g_) and solvent-accessible surface area (SASA) obtained by using both AMBER ff14SB and CHARMM36m (Figure S4) show consistent trends, indicating that oligomers formed by the mutant peptides are more compact than those formed by the wild-type peptides. Second, the mutation exerts similar effects on the time evolution of monomer and oligomer populations (Figures 3 and S5), as well as on π–π stacking interactions (Figures 4 and S6), in both force-field simulations. Third, in both force fields, the mutation has little effect on β-sheet and turn contents but leads to a relative increase in helical population accompanied by a corresponding decrease in the coil content (Figures 5 and S7). Finally, both simulations show that wild-type Aβ_16–22_ peptides preferentially form antiparallel β-sheet arrangements, whereas the K16F/E22F mutant favors mixed β-sheet registries (Figures 7 and S8). Collectively, these results demonstrate that the key conclusions regarding mutation-induced changes in the secondary structure, oligomer compactness, and aggregation pathways are robust and not artifacts of a specific force field.

Aβ_16–22_ is widely used as a minimal model system for amyloid aggregation because it contains the central hydrophobic core critical for full-length Aβ assembly and readily forms well-defined β-sheet fibrils.^48^ Previous experimental and computational studies have established that Aβ_16–22_ aggregation is highly sensitive to sequence perturbations, particularly mutations that alter aromatic interactions, hydrophobic packing, and electrostatic effects.^18–27,52,53,96–103^ For example, Nilsson et al. demonstrated that substitutions at Phe19 and Phe20 modulate aggregation kinetics and thermodynamics through a combined influence of aromaticity, hydrophobicity, and steric effects rather than hydrophobicity alone.^53^ Consistent with this picture, subsequent MD simulations by Berhanu and Hansmann showed that mutations at these positions significantly reshape aggregate stability and morphology, with aromatic-to-aliphatic substitutions either destabilizing or preserving β-sheet assemblies depending on side-chain properties.^97^ In addition to the central hydrophobic cluster, Aβ_16–22_ contains oppositely charged residues at its termini (K16 and E22), which play a key role in stabilizing antiparallel β-sheet architectures. Consistent with previous reports, our simulations show that wild-type Aβ_16–22_ peptides preferentially assemble into antiparallel β-sheet structures. In contrast, the K16F/E22F double mutation profoundly alters this behavior, leading to the formation of mixed β-sheet arrangements rather than a single dominant registry, indicating a substantial reorganization of the oligomerization pathway. This structural shift is quantitatively supported by IIE analysis (Table 1). In the wild-type system, aggregation is primarily driven by electrostatic stabilization, with the intermolecular Coulombic interaction energy (IIEeel = −110 kcal·mol^–1^) substantially exceeding the van der Waals contribution (IIEvdw = −52 kcal·mol^–1^), consistent with the charge-mediated stabilization of antiparallel β-sheets. In contrast, the K16F/E22F mutant exhibits a pronounced reduction in electrostatic interactions (IIEeel = −40 kcal·mol^–1^), accompanied by enhanced van der Waals stabilization (IIEvdw = −76 kcal·mol^–1^), reflecting the substitution of charged residues with phenylalanine, an aromatic amino acid. This redistribution of intermolecular interaction energies shifts the dominant contributions to aggregation from electrostatic interactions toward van der Waals and π–π interactions, consistent with the increased hydrophobicity of the mutated sequence, and is associated with heterogeneous β-sheet registries and mixed β-sheet assemblies. As a result, the double mutation alters the oligomerization pathways of Aβ_16–22_ peptides, leading to distinct secondary-structure populations, modified transition networks, and different aggregate architectures compared to the wild-type system.

## Conclusions

In this work, we investigated the impact of the K16F/E22F double mutation on the oligomerization behavior of Aβ_16–22_ peptides using extensive MD simulations. By employing an optimized simulation framework with multiple independent, long-time scale trajectories, we systematically characterized oligomerization kinetics, secondary structure evolution, and β-sheet assembly pathways. Our results show that the K16F/E22F mutation strengthens both intra- and intermolecular interactions, leading to more compact peptide conformations and accelerated oligomer formation relative to those of the wild-type system.

The mutation also fundamentally alters the oligomerization pathways and β-sheet formation dynamics. In the four-peptide system, aggregation proceeds in a stepwise manner from dimers to tetramers. Wild-type Aβ_16–22_ peptides exhibit frequent interconversions between non−β-sheet and β-sheet states across all oligomeric species, reflecting a highly dynamic assembly process. In contrast, for the K16F/E22F mutant, such structural transitions are largely restricted to the tetrameric state, indicating the enhanced stability of intermediate oligomers. Moreover, wild-type oligomers preferentially form antiparallel β-sheet arrangements, whereas the mutant displays a diverse ensemble of β-sheet registries, including parallel, antiparallel, and mixed configurations, revealing increased structural polymorphism.

## Supplementary Material



## Data Availability

All the data were collected by running MD simulations with AMBER software package.

## Notes

Frequency (%) of secondary structural transitions along the amino acid sequence of wild-type Aβ_16–22_ and K16F/E22F Aβ_16–22_; time evolution of overall structural parameters; distributions of IIE; radius of gyration and solvent-accessible surface area; distributions of the radius of gyration (*R*_g_) and solvent-accessible surface area (SASA) for wild-type and K16F/E22F Aβ_16–22_ peptides obtained from simulations using the AMBER ff14SB and CHARMM36m force fields; and other data from the simulations using CHARMM36m force field (PDF)

## References

[1] Polymeropoulos M. H., Lavedan C., Leroy E. (1997). Mutation in the α-Synuclein Gene Identified in Families with Parkinson’s Disease. Science.

[2] Hardy J., Selkoe D. J. (2002). The Amyloid Hypothesis of Alzheimer’s Disease: Progress and Problems on the Road to Therapeutics. Science.

[3] Singleton A. B., Farrer M., Johnson J. (2003). α-Synuclein Locus Triplication Causes Parkinson’s Disease. Science.

[4] Bloom G. S. (2014). Amyloid-β and Tau: The Trigger and Bullet in Alzheimer Disease Pathogenesis. JAMA Neurol.

[5] Shafiei S. S., Guerrero-Muñoz M. J., Castillo-Carranza D. L. (2017). Tau Oligomers: Cytotoxicity, Propagation, and Mitochondrial Damage. Front. Aging Neurosci..

[6] Cline E. N., Bicca M. A., Viola K. L., Klein W. L. (2018). The Amyloid-β Oligomer Hypothesis: Beginning of the Third Decade. J. Alzheimer’s Dis..

[7] Giuffrida M. L., Caraci F., Pignataro B. (2009). β-Amyloid Monomers Are Neuroprotective. J. Neurosci..

[8] Kamenetz F., Tomita T., Hsieh H. (2003). APP Processing and Synaptic Function. Neuron.

[9] Pearson H. A., Peers C. (2006). Physiological roles for amyloid β peptides. J. Physiol..

[10] Klug G. M. J. A., Losic D., Subasinghe S. S., Aguilar M., Martin L. L., Small D. H. (2003). β-Amyloid protein oligomers induced by metal ions and acid pH are distinct from those generated by slow spontaneous ageing at neutral pH. Eur. J. Biochem..

[11] LeVine H. (2004). Alzheimer’s β-peptide oligomer formation at physiologic concentrations. Anal. Biochem..

[12] Iljina M., Garcia G. A., Dear A. J. (2016). Quantitative analysis of co-oligomer formation by amyloid-beta peptide isoforms. Sci. Rep.

[13] Novo M., Freire S., Al-Soufi W. (2018). Critical aggregation concentration for the formation of early Amyloid-β (1–42) oligomers. Sci. Rep.

[14] Janssen J. C., Beck J. A., Campbell T. A. (2003). Early onset familial Alzheimer’s disease. Neurology.

[15] Chen W. T., Hong C. J., Lin Y. T. (2012). Amyloid-Beta (Aβ) D7H Mutation Increases Oligomeric Aβ42 and Alters Properties of Aβ-Zinc/Copper Assemblies. PLoS One.

[16] Wakutani Y., Watanabe K., Adachi Y. (2004). Novel amyloid precursor protein gene missense mutation (D678N) in probable familial Alzheimer’s disease. J. Neurol Neurosurg Psychiatry.

[17] Zhou L., Brouwers N., Benilova I. (2011). Amyloid precursor protein mutation E682K at the alternative β-secretase cleavage β′-site increases Aβ generation. EMBO Mol. Med..

[18] Kaden D., Harmeier A., Weise C. (2012). Novel APP/Aβ mutation K16N produces highly toxic heteromeric Aβ oligomers. EMBO Mol. Med..

[19] Jiang B., Zhou J., Li H. L., Chen Y. G., Cheng H. R., Ye L. Q., Liu D. S., Chen D. F., Tao Q. Q., Wu Z. Y. (2019). Mutation screening in Chinese patients with familial Alzheimer’s disease by whole-exome sequencing. Neurobiol. Aging.

[20] Yi Y., Xiaobin Y., Hui C. (2020). An APP mutation family exhibiting white matter hyperintensities and cortical calcification in East China. Neurol. Sci..

[21] Obici L., Demarchi A., de Rosa G. (2005). A novel AβPP mutation exclusively associated with cerebral amyloid angiopathy. Ann. Neurol..

[22] Hendriks L., van Duijn C. M., Cras P. (1992). Presenile dementia and cerebral haemorrhage linked to a mutation at codon 692 of the β–amyloid precursor protein gene. Nat. Genet..

[23] Levy E., Carman M. D., Fernandez-Madrid I. J. (1990). Mutation of the Alzheimer’s Disease Amyloid Gene in Hereditary Cerebral Hemorrhage, Dutch Type. Science.

[24] Van Broeckhoven C., Haan J., Bakker E. (1990). Amyloid β Protein Precursor Gene and Hereditary Cerebral Hemorrhage with Amyloidosis (Dutch). Science.

[25] Rossi G., Macchi G., Porro M. (1998). Fatal familial insomnia. Neurology.

[26] Nilsberth C., Westlind-Danielsson A., Eckman C. B. (2001). The “Arctic” APP mutation (E693G) causes Alzheimer’s disease by enhanced Aβ protofibril formation. Nat. Neurosci..

[27] Ovchinnikova O. Y., Finder V. H., Vodopivec I., Nitsch R. M., Glockshuber R. (2011). The Osaka FAD Mutation E22Δ Leads to the Formation of a Previously Unknown Type of Amyloid β Fibrils and Modulates Aβ Neurotoxicity. J. Mol. Biol..

[28] Grabowski T. J., Cho H. S., Vonsattel J. P., Rebeck G. W., Greenberg S. M. (2001). Novel amyloid precursor protein mutation in an Iowa family with dementia and severe cerebral amyloid angiopathy. Ann. Neurol..

[29] Seuma M., Lehner B., Bolognesi B. (2022). An atlas of amyloid aggregation: the impact of substitutions, insertions, deletions and truncations on amyloid beta fibril nucleation. Nat. Commun..

[30] Jorgensen W. L., Maxwell D. S., Tirado-Rives J. (1996). Development and Testing of the OPLS All-Atom Force Field on Conformational Energetics and Properties of Organic Liquids. J. Am. Chem. Soc..

[31] Pearlman D. A., Case D. A., Caldwell J. W. (1995). AMBER, a package of computer programs for applying molecular mechanics, normal mode analysis, molecular dynamics and free energy calculations to simulate the structural and energetic properties of molecules. Comput. Phys. Commun..

[32] Kalé L., Skeel R., Bhandarkar M. (1999). NAMD2: Greater Scalability for Parallel Molecular Dynamics. J. Comput. Phys..

[33] Christen M., Hünenberger P. H., Bakowies D. (2005). The GROMOS software for biomolecular simulation: GROMOS05. J. Comput. Chem..

[34] Brooks B. R., Brooks C. L., Mackerell A. D., Nilsson L., Petrella R. J., Roux B., Won Y., Archontis G., Bartels C., Boresch S. (2009). CHARMM: The biomolecular simulation program. J. Comput. Chem..

[35] Ponder J. W., Wu C., Ren P. (2010). Current Status of the AMOEBA Polarizable Force Field. J. Phys. Chem. B.

[36] Hu Z., Jiang J. (2010). Assessment of biomolecular force fields for molecular dynamics simulations in a protein crystal. J. Comput. Chem..

[37] Schmid N., Christ C. D., Christen M., Eichenberger A. P., van Gunsteren W. F. (2012). Architecture, implementation and parallelisation of the GROMOS software for biomolecular simulation. Comput. Phys. Commun..

[38] Wang W., Donini O., Reyes C. M., Kollman P. A. (2001). Biomolecular Simulations: Recent Developments in Force Fields, Simulations of Enzyme Catalysis, Protein-Ligand, Protein-Protein, and Protein-Nucleic Acid Noncovalent Interactions. Annu. Rev. Biophys. Biomol. Struct..

[39] Brooks C. L. (2002). Protein and Peptide Folding Explored with Molecular Simulations. Acc. Chem. Res..

[40] Andrec M., Felts A. K., Gallicchio E., Levy R. M. (2005). Protein folding pathways from replica exchange simulations and a kinetic network model. Proc. Natl. Acad. Sci..

[41] Shaw D. E., Maragakis P., Lindorff-Larsen K. (2010). Atomic-Level Characterization of the Structural Dynamics of Proteins. Science.

[42] Wray R., Iscla I., Gao Y., Li H., Wang J., Blount P. (2016). Dihydrostreptomycin Directly Binds to, Modulates, and Passes through the MscL Channel Pore. PLoS Biol..

[43] Barz B., Liao Q., Strodel B. (2018). Pathways of Amyloid-β Aggregation Depend on Oligomer Shape. J. Am. Chem. Soc..

[44] Man V. H., Nguyen P. H., Derreumaux P. (2017). High-Resolution Structures of the Amyloid-β 1–42 Dimers from the Comparison of Four Atomistic Force Fields. J. Phys. Chem. B.

[45] Nasica-Labouze J., Nguyen P. H., Sterpone F. (2015). Amyloid β Protein and Alzheimer’s Disease: When Computer Simulations Complement Experimental Studies. Chem. Rev..

[46] Carballo-Pacheco M., Ismail A. E., Strodel B. (2018). On the Applicability of Force Fields To Study the Aggregation of Amyloidogenic Peptides Using Molecular Dynamics Simulations. J. Chem. Theory Comput..

[47] Man V. H., He X., Derreumaux P. (2019). Effects of All-Atom Molecular Mechanics Force Fields on Amyloid Peptide Assembly: The Case of Aβ16–22 Dimer. J. Chem. Theory Comput..

[48] Balbach J. J., Ishii Y., Antzutkin O. N. (2000). Amyloid Fibril Formation by Aβ16–22, a Seven-Residue Fragment of the Alzheimer’s β-Amyloid Peptide, and Structural Characterization by Solid State NMR. Biochemistry.

[49] Ma B., Nussinov R. (2006). Simulations as analytical tools to understand protein aggregation and predict amyloid conformation. Curr. Opin. Chem. Biol..

[50] Nguyen P. H., Li M. S., Stock G., Straub J. E., Thirumalai D. (2007). Monomer adds to preformed structured oligomers of Aβ-peptides by a two-stage dock–lock mechanism. Proc. Natl. Acad. Sci..

[51] Tao K., Wang J., Zhou P. (2011). Self-Assembly of Short Aβ(16–22) Peptides: Effect of Terminal Capping and the Role of Electrostatic Interaction. Langmuir.

[52] Senguen F. T., Doran T. M., Anderson E. A., Nilsson B. L. (2010). Clarifying the influence of core amino acid hydrophobicity, secondary structure propensity, and molecular volume on amyloid-β 16–22 self-assembly. Mol. Biosyst.

[53] Senguen F. T., Lee N. R., Gu X. (2010). Probing aromatic, hydrophobic, and steric effects on the self-assembly of an amyloid-β fragment peptide. Mol. Biosyst.

[54] Man V. H., He X., Wang J. (2022). Stable Cavitation Interferes with Aβ16–22 Oligomerization. J. Chem. Inf. Model..

[55] Petkova A. T., Buntkowsky G., Dyda F., Leapman R. D., Yau W. M., Tycko R. (2004). Solid State NMR Reveals a pH-dependent Antiparallel β-Sheet Registry in Fibrils Formed by a β-Amyloid Peptide. J. Mol. Biol..

[56] Poulsen S. A., Watson A. A., Fairlie D. P., Craik D. J. (2000). Solution Structures in Aqueous SDS Micelles of Two Amyloid β Peptides of Aβ(1–28) Mutated at the α-Secretase Cleavage Site (K16E, K16F). J. Struct. Biol..

[57] de Groot N. S., Aviles F. X., Vendrell J., Ventura S. (2006). Mutagenesis of the central hydrophobic cluster in Aβ42 Alzheimer’s peptide. FEBS J..

[58] Koch M., Enzlein T., Chen S. (2023). APP substrate ectodomain defines amyloid-β peptide length by restraining γ-secretase processivity and facilitating product release. EMBO J..

[59] Man V. H., He X., Ji B., Liu S., Xie X. Q., Wang J. (2019). Molecular Mechanism and Kinetics of Amyloid-β42 Aggregate Formation: A Simulation Study. ACS Chem. Neurosci..

[60] Ono K., Condron M. M., Teplow D. B. (2009). Structure–neurotoxicity relationships of amyloid β-protein oligomers. Proc. Natl. Acad. Sci..

[61] Jana M. K., Cappai R., Pham C. L. L., Ciccotosto G. D. (2016). Membrane-bound tetramer and trimer Aβ oligomeric species correlate with toxicity towards cultured neurons. J. Neurochem..

[62] Case, D. A. ; Walker, R. C. ; Cheatham, T. E., III. ; AMBER 19; University of California: San Francisco, 2019.

[63] Maier J. A., Martinez C., Kasavajhala K., Wickstrom L., Hauser K. E., Simmerling C. (2015). ff14SB: Improving the Accuracy of Protein Side Chain and Backbone Parameters from ff99SB. J. Chem. Theory Comput..

[64] Jorgensen W. L., Chandrasekhar J., Madura J. D., Impey R. W., Klein M. L. (1983). Comparison of simple potential functions for simulating liquid water. J. Chem. Phys..

[65] Essmann U., Perera L., Berkowitz M. L., Darden T., Lee H., Pedersen L. G. (1995). A smooth particle mesh Ewald method. J. Chem. Phys..

[66] Berendsen H. J. C., Postma J. P. M., van Gunsteren W. F., DiNola A., Haak J. R. (1984). Molecular dynamics with coupling to an external bath. J. Chem. Phys..

[67] Forester T. R., Smith W. (1998). SHAKE, rattle, and roll: Efficient constraint algorithms for linked rigid bodies. J. Comput. Chem..

[68] Frishman D., Argos P. (1995). Knowledge-based protein secondary structure assignment. Proteins Struct Funct Bioinf..

[69] Heinig M., Frishman D. (2004). STRIDE: a web server for secondary structure assignment from known atomic coordinates of proteins. Nucleic Acids Res..

[70] Roe D. R., Cheatham T. E. I. (2013). PTRAJ and CPPTRAJ: Software for Processing and Analysis of Molecular Dynamics Trajectory Data. J. Chem. Theory Comput..

[71] Weiser J., Shenkin P. S., Still W. C. (1999). Approximate atomic surfaces from linear combinations of pairwise overlaps (LCPO). J. Comput. Chem..

[72] Sugita Y., Okamoto Y. (1999). Replica-exchange molecular dynamics method for protein folding. Chem. Phys. Lett..

[73] Zhang T., Nguyen P. H., Nasica-Labouze J., Mu Y., Derreumaux P. (2015). Folding Atomistic Proteins in Explicit Solvent Using Simulated Tempering. J. Phys. Chem. B.

[74] Cukalevski R., Boland B., Frohm B., Thulin E., Walsh D., Linse S. (2012). Role of Aromatic Side Chains in Amyloid β-Protein Aggregation. ACS Chem. Neurosci..

[75] Liu D., Fu D., Zhang L., Sun L. (2021). Detection of amyloid-beta by Fmoc-KLVFF self-assembled fluorescent nanoparticles for Alzheimer’s disease diagnosis. Chin. Chem. Lett..

[76] Press-Sandler O., Miller Y. (2022). Molecular insights into the primary nucleation of polymorphic amyloid β dimers in DOPC lipid bilayer membrane. Protein Sci..

[77] Ladiwala A. R. A., Dordick J. S., Tessier P. M. (2011). Aromatic Small Molecules Remodel Toxic Soluble Oligomers of Amyloid β through Three Independent Pathways. J. Biol. Chem..

[78] Irwin J. A., Edward Wong H., Kwon I. (2015). Determining binding sites of polycyclic aromatic small molecule-based amyloid-beta peptide aggregation modulators using sequence-specific antibodies. Anal. Biochem..

[79] Stanković I. M., Niu S., Hall M. B., Zarić S. D. (2020). Role of aromatic amino acids in amyloid self-assembly. Int. J. Biol. Macromol..

[80] Onufriev A., Bashford D., Case D. A. (2004). Exploring protein native states and large-scale conformational changes with a modified generalized born model. Proteins: Struct., Funct., Bioinf..

[81] Favrin G., Irbäck A., Mohanty S. (2004). Oligomerization of Amyloid Aβ16–22 Peptides Using Hydrogen Bonds and Hydrophobicity Forces. Biophys. J..

[82] Klimov D. K., Thirumalai D. (2003). Dissecting the Assembly of Aβ16–22 Amyloid Peptides into Antiparallel β Sheets. Structure.

[83] Gazit E. (2002). A possible role for π-stacking in the self-assembly of amyloid fibrils. FASEB J..

[84] Cheon M., Chang I., Hall C. K. (2011). Spontaneous Formation of Twisted Aβ16–22 Fibrils in Large-Scale Molecular-Dynamics Simulations. Biophys. J..

[85] Wu C., Biancalana M., Koide S., Shea J. E. (2009). Binding Modes of Thioflavin-T to the Single-Layer β-Sheet of the Peptide Self-Assembly Mimics. J. Mol. Biol..

[86] Jarvet J., Damberg P., Bodell K., Göran Eriksson L. E., Gräslund A. (2000). Reversible Random Coil to β-Sheet Transition and the Early Stage of Aggregation of the Aβ(12–28) Fragment from the Alzheimer Peptide. J. Am. Chem. Soc..

[87] Fatafta H., Khaled M., Owen M. C., Sayyed-Ahmad A., Strodel B. (2021). Amyloid-β peptide dimers undergo a random coil to β-sheet transition in the aqueous phase but not at the neuronal membrane. Proc. Natl. Acad. Sci..

[88] Xu Y., Shen J., Luo X. (2005). Conformational transition of amyloid β-peptide. Proc. Natl. Acad. Sci..

[89] Straub J. E., Thirumalai D. (2010). Principles governing oligomer formation in amyloidogenic peptides. Curr. Opin. Struct. Biol..

[90] Cheon M., Hall C. K., Chang I. (2015). Structural Conversion of Aβ17–42 Peptides from Disordered Oligomers to U-Shape Protofilaments via Multiple Kinetic Pathways. PLoS Comput. Biol..

[91] Misra P., Kodali R., Chemuru S., Kar K., Wetzel R. (2016). Rapid α-oligomer formation mediated by the Aβ C terminus initiates an amyloid assembly pathway. Nat. Commun..

[92] Man V. H., He X., Gao J., Wang J. (2021). Effects of All-Atom Molecular Mechanics Force Fields on Amyloid Peptide Assembly: The Case of PHF6 Peptide of Tau Protein. J. Chem. Theory Comput..

[93] Ponder J. W., Case D. A. (2003). Force Fields for Protein Simulations. Adv. Protein Chem..

[94] Huang J., Rauscher S., Nawrocki G. (2017). CHARMM36m: an improved force field for folded and intrinsically disordered proteins. Nat. Methods.

[95] Coppa C., Bazzoli A., Barkhordari M., Contini A. (2023). Accelerated Molecular Dynamics for Peptide Folding: Benchmarking Different Combinations of Force Fields and Explicit Solvent Models. J. Chem. Inf. Model..

[96] Miravalle L., Tokuda T., Chiarle R. (2000). Substitutions at Codon 22 of Alzheimer’s Aβ Peptide Induce Diverse Conformational Changes and Apoptotic Effects in Human Cerebral Endothelial Cells. J. Biol. Chem..

[97] Berhanu W. M., Hansmann U. H. E. (2012). Side-chain hydrophobicity and the stability of Aβ_16–22_ aggregates. Protein Sci..

[98] Petty S. A., Decatur S. M. (2005). Experimental Evidence for the Reorganization of β-Strands within Aggregates of the Aβ_(16–22)_ Peptide. J. Am. Chem. Soc..

[99] Wang J., Gülich S., Bradford C., Ramirez-Alvarado M., Regan L. (2005). A Twisted Four-Sheeted Model for an Amyloid Fibril. Structure.

[100] Mehta A. K., Lu K., Childers W. S. (2008). Facial Symmetry in Protein Self-Assembly. J. Am. Chem. Soc..

[101] Liang C., Ni R., Smith J. E., Childers W. S., Mehta A. K., Lynn D. G. (2014). Kinetic Intermediates in Amyloid Assembly. J. Am. Chem. Soc..

[102] Smith J. E., Liang C., Tseng M. (2015). Defining the Dynamic Conformational Networks of Cross-β Peptide Assembly. Isr. J. Chem..

[103] Li X., Lei J., Qi R., Xie L., Wei G. (2019). Mechanistic insight into E22Q-mutation-induced antiparallel-to-parallel β-sheet transition of Aβ16–22 fibrils: an all-atom simulation study. Phys. Chem. Chem. Phys..

