# Supplementary material for: K16F/E22F Mutation Promotes Oligomerization and Alters β‑Sheet Topology of Aβ16–22 Peptides: Insights from Molecular Dynamics Simulations
Source: ACS Chem Neurosci. 2026 Mar 23;17(7):1387–400. doi: 10.1021/acschemneuro.6c00003 (PMC13056258; doi:10.1021/acschemneuro.6c00003)
Supplement: Supplementary file 1 [file cn6c00003_si_001.pdf]

SUPPORTING INFORMATION

K16F/E22F Mutation Promotes Oligomerization and Alters β-Sheet Topology of Aβ16–22 Peptides: Insights from Molecular Dynamics Simulations

Viet Hoang Man,[1*] Xibing He,[1] Taoyu Niu,[1] Lianjin Cai,[1] Fengyang Han,[1] Phuong Nguyen,[2] and Junmei Wang[1*]

[1] *Department of Pharmaceutical Sciences and Computational Chemical Genomics Screening Center, School of Pharmacy, University of Pittsburgh, Pittsburgh, PA 15261, USA.*

[2] *Universite Paris Cite, CNRS, Laboratoire de Biochimie Theorique, 13 rue Pierre et Marie Curie, 75005 Paris, France.*

*E-Mails:

Viet Hoang Man: vhm3@pitt.edu
Junmei Wang: junmei.wang@pitt.edu

Table S1: The frequency (%) of secondary structural transitions along the amino acid sequence of wild-type Aβ_{16-22}. **E**, **H**, **T** and **C** represent for β-sheet, helix, turn and coil structures, respectively.

Res	E→H	H→E	E→T	T→E	E→C	C→E	H→T	T→H	H→C	C→H	T→C	C→T
**K**	0	0	0.50	0.50	0.91	0.91	1.20	1.20	1.32	1.32	11.72	11.73
**L**	0	0	1.87	1.87	1.67	1.67	5.67	5.67	1.04	1.04	13.93	13.93
**V**	0	0	1.59	1.59	1.86	1.86	12.82	12.82	0.19	0.19	10.61	10.62
**F**	0	0	1.55	1.55	1.72	1.72	14.26	14.26	0.01	0.01	8.35	8.35
**F**	0	0	1.74	1.74	1.39	1.4	11.76	11.75	1.81	1.82	13.05	13.04
**A**	0	0	0.82	0.82	1.17	1.16	4.29	4.29	2.36	2.36	19.07	19.07
**E**	0	0	0.23	0.23	0.37	0.37	1.39	1.40	2.49	2.48	10.63	10.64

Table S2: The frequency (%) of secondary structural transitions along the amino acid sequence of K16F/E22F Aβ_{16-22}. **E**, **H**, **T** and **C** represent for β-sheet, helix, turn and coil structures, respectively.

Res	E→H	H→E	E→T	T→E	E→C	C→E	H→T	T→H	H→C	C→H	T→C	C→T
**F**	0	0	0.33	0.33	0.78	0.78	2.78	2.76	2.82	2.83	15.8	15.78
**L**	0	0	1.61	1.61	1.35	1.35	9.77	9.77	1.95	1.95	13.28	13.28
**V**	0	0	1.75	1.75	1.55	1.55	15.5	15.49	0.37	0.37	9.19	9.18
**F**	0	0	1.59	1.59	1.42	1.42	17.71	17.71	0.01	0.01	6.39	6.39
**F**	0	0	1.65	1.65	1.18	1.18	13.74	13.74	2.71	2.71	14.17	14.17
**A**	0	0	0.92	0.92	1.17	1.17	7.28	7.28	3.84	3.85	20.17	20.16
**F**	0	0	0.31	0.31	0.38	0.38	2.21	2.20	4.40	4.40	15.74	15.73

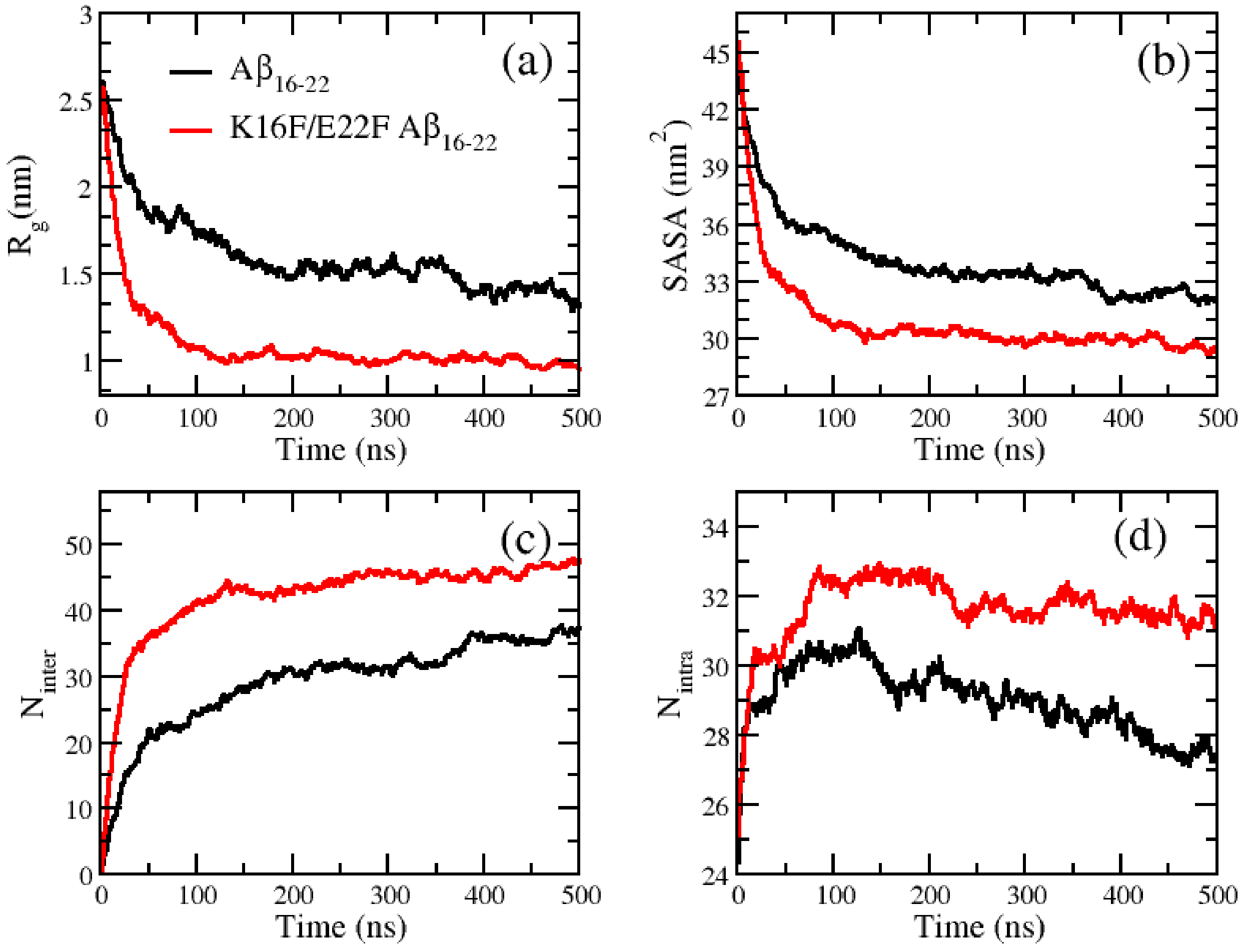

Figure S1: Time evolution of radius of gyration-R_g (a), solvent accessible surface area-SASA (b), number of intermolecular residue-residue interaction-N_{inter} (c), number of intramolecular residue-residue interaction-N_{intra} (d).

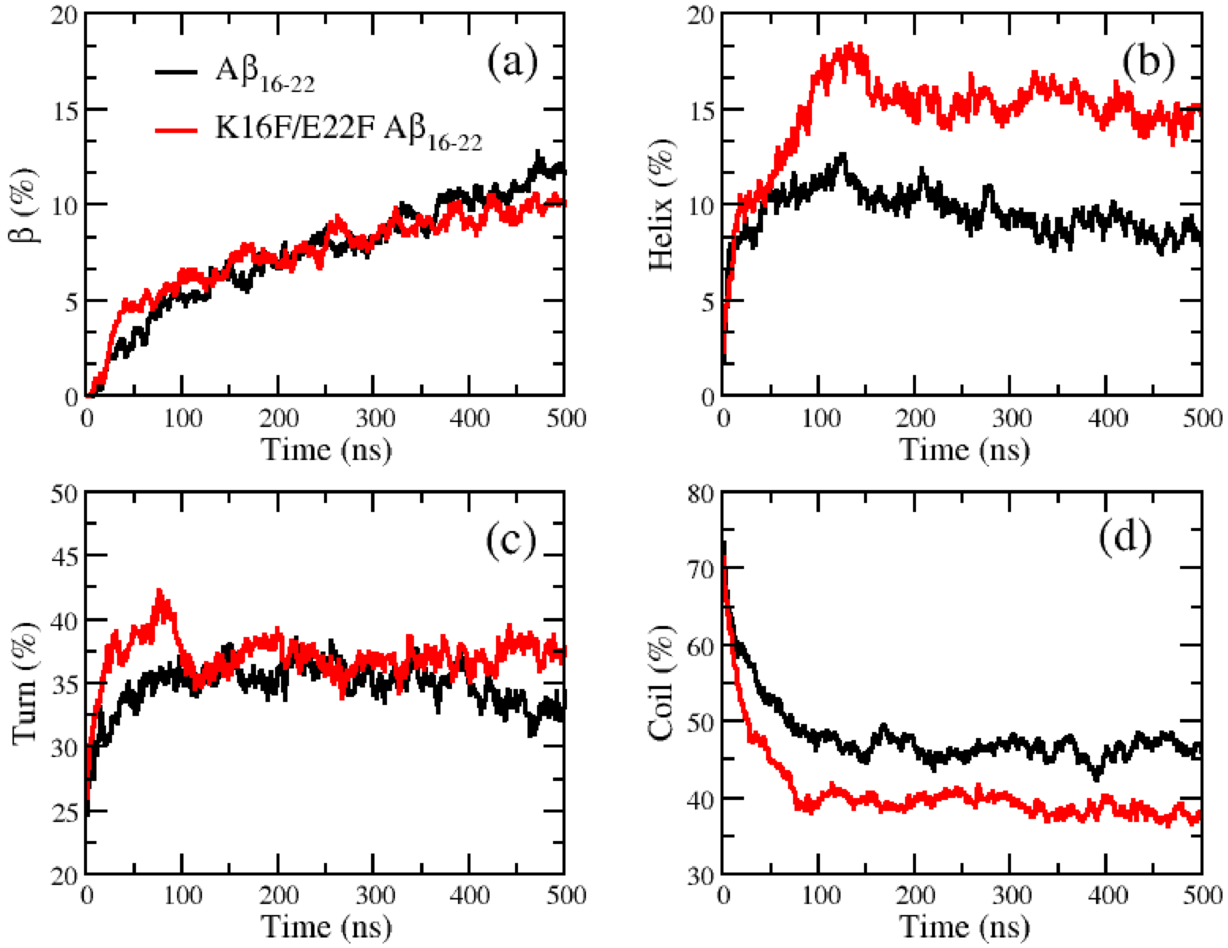

Figure S2: Time evolution of secondary structure contents, including β-sheet (a), helix (b), turn (c), and coil (d).

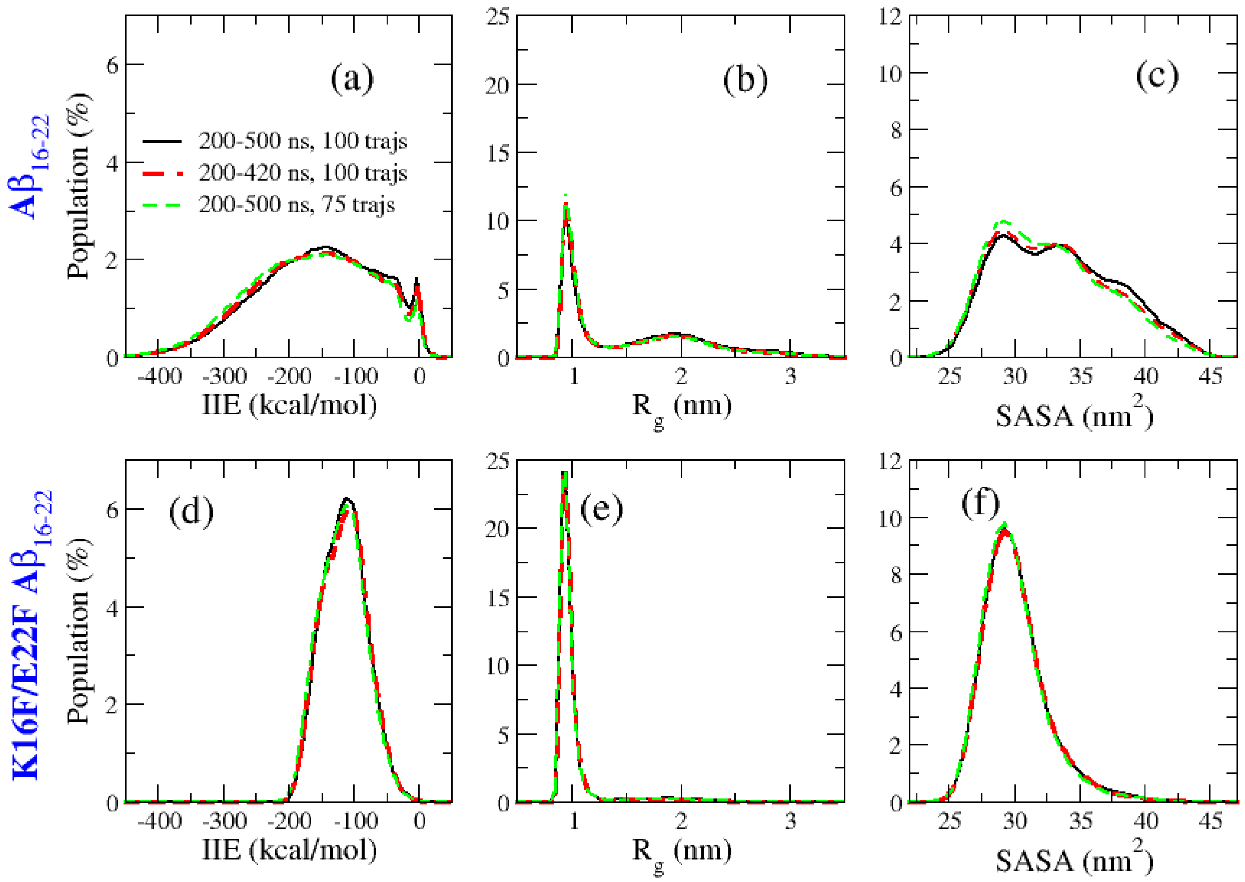

Figure S3: Distributions of intermolecular interaction energy (IIE), radius of gyration (R_g) and solvent accessible surface area (SASA) in the wild-type (a, b and c panels) and mutated (d, e and f) systems. The distributions were generated using three conformation ensembles: all snapshots in the last 300 ns of 100 500-ns MD trajectories (black lines), snapshots sampled from 200 ns to 420 ns of 100 500-ns MD trajectories (red dashed lines); and last 300 ns of 75 randomly selected 500-ns MD trajectories (green dashed lines).

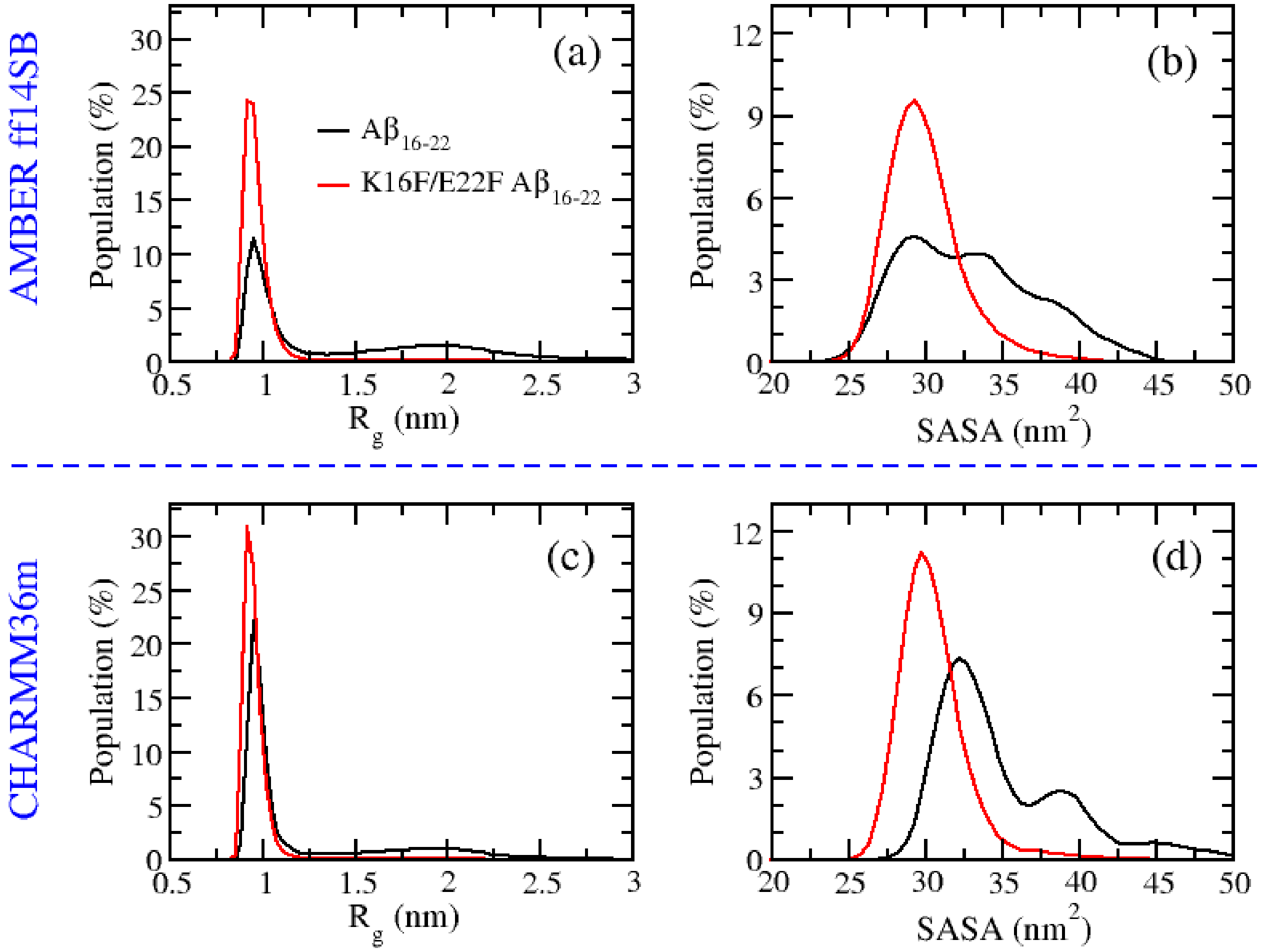

Figure S4: Distributions of the radius of gyration (R_g) and solvent-accessible surface area (SASA) for wild-type and K16F/E22F Aβ_{16-22} peptides obtained from simulations using the AMBER ff14SB and CHARMM36m force fields. Data for the wild-type and mutant systems are shown in black and red, respectively. Results from the AMBER ff14SB simulations are shown in panels (a) and (b), while those from the CHARMM36m simulations are shown in panels (c) and (d).

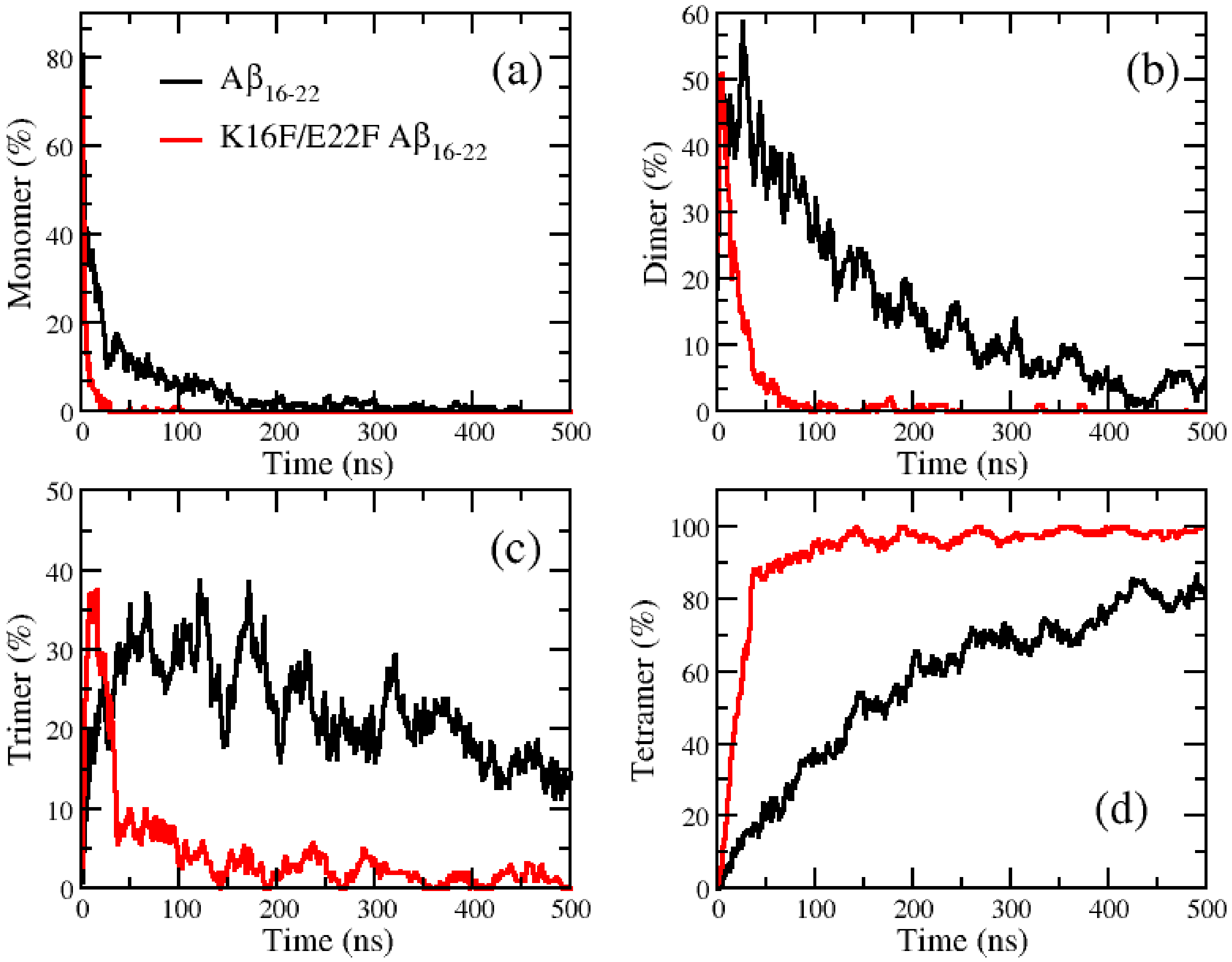

Figure S5: Time evolution of the populations of monomers (a), dimers (b), trimers (c), and tetramers (d) for the wild-type (black lines) and K16F/E22F mutant (red lines) $A\beta_{16-22}$ peptides from the simulations with Charmm36m force field.

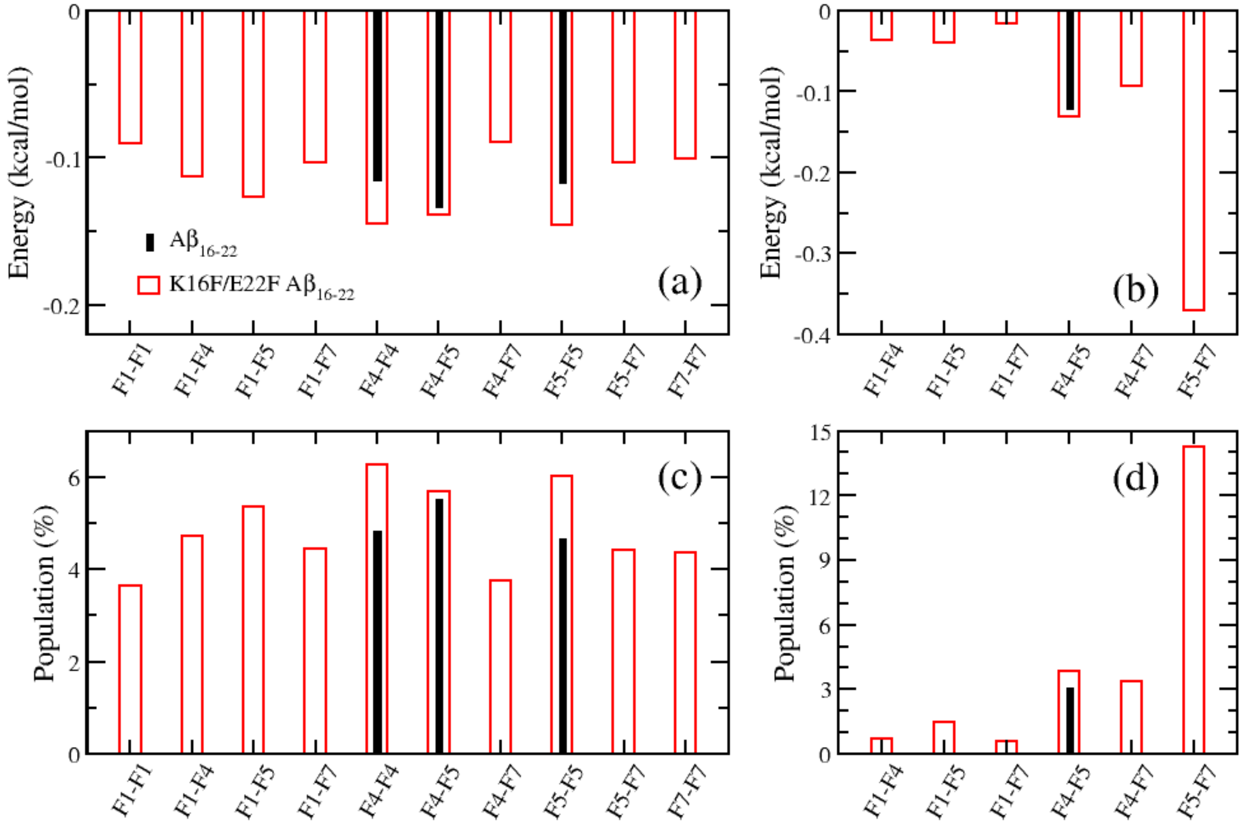

Figure S6. The π–π interactions in Aβ16-22 peptides from the simulations with Charmm36m force field. Shown are the time-averaged interaction energies between pairs of phenylalanine residues (a, b) and the corresponding populations of π–π interactions (c, d). Panels (a) and (c) correspond to intermolecular phenylalanine interactions, whereas panels (b) and (d) correspond to intramolecular phenylalanine interactions. The interaction population is defined as the percentage of simulation frames in which the instantaneous π–π interaction energy between a given phenylalanine pair is lower than −1.0 kcal/mol, whereas the energies shown in panels (a) and (b) represent averages over the full trajectories and therefore span a narrower energy range. Black and red bars denote the wild-type and K16F/E22F mutant $A\beta_{16\text{-}22}$ peptides, respectively.

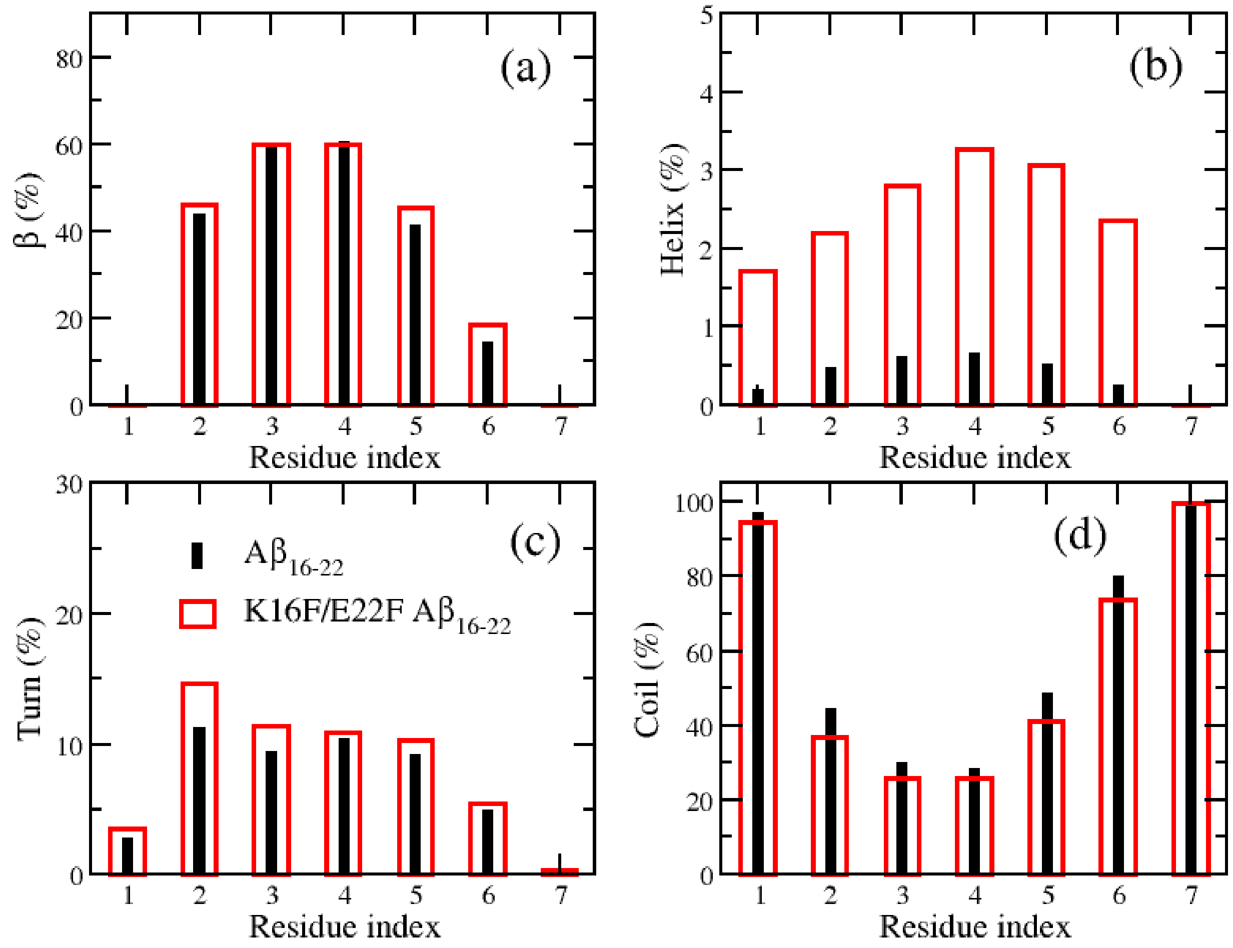

Figure S7: Secondary structural populations along the amino acid sequence of A$\beta_{16\text{-}22}$ peptides from the simulations with Charmm36m force field. The data were calculated using snapshots sampled in the last 300 ns of all 100 MD trajectories for each system.

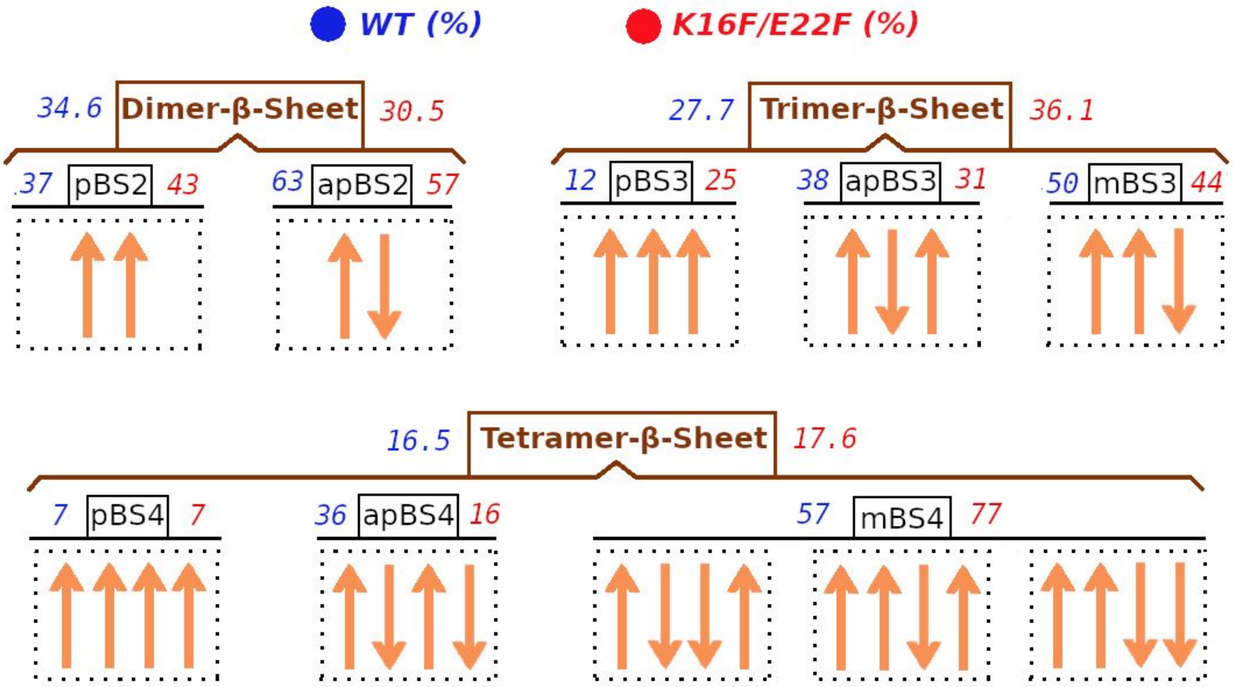

Figure S8: The populations of different β-sheet types, which include parallel dimer β-sheet (pBS2), anti-parallel dimer β-sheet (apBS2), parallel trimer β-sheet (pBS3), anti-parallel trimer β-sheet (apBS3), mix-trimer β-sheet (mBS3), parallel tetramer β-sheet (pBS4), anti-parallel tetramer β-sheet (apBS4), mix-tetramer β-sheet (mBS4). The data were generated using snapshots collected from the 500 ns of all 100 MD trajectories for each system from the simulations with Charmm36m force field.
